# Novel Role of 3’UTR-Embedded Alu Elements as Facilitators of Processed Pseudogene Genesis and Host Gene Capture by Viral Genomes

**DOI:** 10.1371/journal.pone.0169196

**Published:** 2016-12-29

**Authors:** Domènec Farré, Pablo Engel, Ana Angulo

**Affiliations:** 1 Immunology Unit, Department of Biomedical Sciences, Medical School, University of Barcelona, Barcelona, Spain; 2 Institut d’Investigacions Biomèdiques August Pi i Sunyer, Barcelona, Spain; University of Muenster, GERMANY

## Abstract

Since the discovery of the high abundance of Alu elements in the human genome, the interest for the functional significance of these retrotransposons has been increasing. Primate Alu and rodent Alu-like elements are retrotransposed by a mechanism driven by the LINE1 (L1) encoded proteins, the same machinery that generates the L1 repeats, the processed pseudogenes (PPs), and other retroelements. Apart from free Alu RNAs, Alus are also transcribed and retrotranscribed as part of cellular gene transcripts, generally embedded inside 3’ untranslated regions (UTRs). Despite different proposed hypotheses, the functional implication of the presence of Alus inside 3’UTRs remains elusive. In this study we hypothesized that Alu elements in 3’UTRs could be involved in the genesis of PPs. By analyzing human genome data we discovered that the existence of 3’UTR-embedded Alu elements is overrepresented in genes source of PPs. In contrast, the presence of other retrotransposable elements in 3’UTRs does not show this PP linked overrepresentation. This research was extended to mouse and rat genomes and the results accordingly reveal overrepresentation of 3’UTR-embedded B1 (Alu-like) elements in PP parent genes. Interestingly, we also demonstrated that the overrepresentation of 3’UTR-embedded Alus is particularly significant in PP parent genes with low germline gene expression level. Finally, we provide data that support the hypothesis that the L1 machinery is also the system that herpesviruses, and possibly other large DNA viruses, use to capture host genes expressed in germline or somatic cells. Altogether our results suggest a novel role for Alu or Alu-like elements inside 3’UTRs as facilitators of the genesis of PPs, particularly in lowly expressed genes. Moreover, we propose that this L1-driven mechanism, aided by the presence of 3’UTR-embedded Alus, may also be exploited by DNA viruses to incorporate host genes to their viral genomes.

## Introduction

Alu elements are the most abundant repetitive elements in the human genome; with 1.1 million copies, they represent about 10% of the genome [1, 2]. They have a length of approximately 300 bp and a dimeric structure, with two similar but distinct monomers joined by an A-rich linker and followed by a short poly(A) tail. These kinds of short interspersed nucleotide elements (SINEs) are retrotransposons specific to primates. However, rodent genomes have other SINEs, named B1 elements, which are Alu-like elements with a monomeric structure and a length of approximately 140 bp [3]. Interestingly, old free Alu monomers, which predate the first dimeric element, are still present in primate genomes [4, 5]. Phylogenetic studies indicate that the monomers of Alu and the B1 elements originated from the gene that encodes the 7SL RNA, the RNA component of the signal recognition particle (SRP), which is the ribonucleoprotein that targets secreted proteins to the endoplasmic reticulum [3–6]. Rodent genomes have in addition B2 and ID elements, which are tRNA-derived SINEs, and B4 elements, which resemble a fusion between B1 and ID elements. The total number of copies of B1, B2, B4, and ID elements in mouse (1.4 millions) surpasses that of human Alu elements [7]. Both primates and rodents have also MIR (mammalian-wide interspersed repeat) elements, which are ancient tRNA-derived SINEs.

SINEs lack protein-coding capability and, since the 1990s, it had been hypothesized that their retrotransposition is driven by long interspersed nucleotide elements (LINEs), retrotransposons that are transcribed by RNA polymerase II (Pol II) and encode the enzymes required for their mobility [8]. Subsequently, *in vitro* retrotransposition of Alu, B1, and B2 elements mediated by L1 (or LINE1), a LINE present in all mammals, was formally demonstrated [9, 10]. L1 is the only currently active autonomous transposon in humans [2, 11–18]. L1 elements have two open reading frames (ORF1 and ORF2) that encode two proteins critical for the process of retrotransposition. Whereas the role of ORF1 is still poorly understood, it is known that the protein encoded by ORF2 (ORF2p) is an endonuclease and reverse transcriptase enzyme that nicks the DNA and reverse transcribes the L1 RNA into the nicked site [19–22]. Mammalian SINEs such as Alus in primates and B1s, B2s, and IDs in rodents share the insertion site motif 5’-TT/AAAA-3’, the same motif recognized by L1 ORF2p for new L1 insertions [8–10, 20, 23].

L1 elements have *cis*-preference, more efficiently promoting the reverse transcription of their own messenger RNAs (mRNAs); this effect is explained by the spatial proximity during translation between the nascent L1 proteins and the mRNA that encodes them [24–27]. Any other RNA shows a much lower level of retrotransposition mediated by L1. However, Alu elements display a high rate of retrotransposition, bypassing the *cis*-preference of L1 [9]. As Alu RNAs can bind the cognate SRP proteins SRP9 and SRP14 (SRP9/14 heterodimer) *in vitro* and *in vivo* [28–31], this specific association has been proposed as a way to localize the Alu RNA to the ribosome where it is hypothesized to interact with the nascent ORF2 protein of L1, increasing Alu retrotransposition efficiency [9, 24].

In normal conditions, the internal RNA polymerase III (Pol III) promoter of Alu elements is not sufficient to drive transcription *in vivo* and very few Alu elements of the genome are able to retrotranspose [32–34]. However, the expression of free Alu RNAs by Pol III increases up to 20-fold under various stress conditions, such as heat shock or viral infection, concomitant with the rise of LINE1 expression [35–38]. Apart from these Pol III-transcribed free Alu RNAs, Alu elements integrated inside genes, named “embedded Alu RNAs”, are also transcribed as part of protein and non-protein coding transcripts by Pol II [39]. Actually, the majority of Alu-containing RNAs detected in HeLa cells and other cell lines are transcribed from non-Alu promoters [36]; in other words, they correspond to embedded Alus. It has been also observed that, inside mRNAs, there is a tendency to accumulate Alu elements in 3’ untranslated regions (UTRs) [39]. Several roles have been assigned to these 3’UTR-embedded Alus, such as regulators of mRNA stability [40] or microRNA targets that could affect gene expression [41], but their functional importance is still not clear.

Besides L1s and SINEs, processed pseudogenes (PPs) are generated using the L1 machinery too [26, 42, 43], but at a much lower frequency than Alu and L1 elements. Also known as retropseudogenes or retrocopies, processed pseudogenes are the product of retrotranscription from cellular mRNAs. Whereas they lack introns and the 5’ promoter sequence, they have a poly(A) tail at their 3’ end and are flanked by direct repeats (target site duplications). Approximately 8,000 PPs have been identified in the human genome reference sequence [44–46], though last GENCODE releases contain more that 10,000 PPs annotated [47]. The functional gene with the greatest sequence similarity to a pseudogene is considered the parent gene (alternatively called source gene). Most parent genes have just one pseudogene, but some are associated with a large number of pseudogenes [47]. It is well known that housekeeping genes tend to have more processed pseudogenes [46, 48]. However, it is not completely understood why some genes are prone to generate pseudogenes, and in particular processed pseudogenes, while others not.

In another field, genomes of large DNA viruses, in particular herpesviruses, contain open reading frames that exhibit evident sequence similarities to host genes [49–52]. These homologous genes, which can account for more than 30% of the viral coding potential [53], mostly encode proteins involved in direct interaction with the host that are not essential for *in vitro* viral replication. A large number of these viral homologs are implicated in immune defense, but there are also proteins involved in apoptosis, cell cycle regulation, or nucleic acid metabolism [49–55]. Thus, the study of the genesis of these viral genes may be of great importance to better understand the mechanisms of viral evolutionary adaptation and pathogenesis. It is assumed that these genes have been captured by the virus from the host genome during million of years of co-evolution and this kind of gene piracy is regarded as an important evolutive viral strategy. However, the mechanism of horizontal gene transfer to explain the origin of these viral genes is still poorly known. Some viral captured genes conserve the intron structure of the parent host gene, suggesting that they were created by direct recombination between the viral and host genomes, but most viral homologs are intronless. It was postulated that they originated from host spliced transcripts by a procedure that involves retroviruses [56]. Although this hypothesis has been widely accepted, evidences supporting it have not been provided.

In the present study we hypothesized that the presence of Alu elements in 3’UTRs affects the genesis of processed pseudogenes. Accordingly, we discovered that there is a significant overrepresentation of Alu or Alu-like elements, and no other retrotransposons, in the 3’UTRs of human or rodent genes that generated processed pseudogenes, especially genes with low expression. These results suggest a novel role of Alus: 3’UTR-embedded Alu or Alu-like elements facilitate the genesis of processed pseudogenes by L1 products. Moreover, we found that most primate genes captured by herpesviruses have Alus in their 3’UTRs. Thus, we propose that herpesviruses use the same L1 driven host mechanism that generates processed pseudogenes in order to incorporate host genes to their viral genomes.

## Results

### The presence of 3’UTR-embedded Alu elements is overrepresented in genes parent of processed pseudogenes

**Fig 1:**
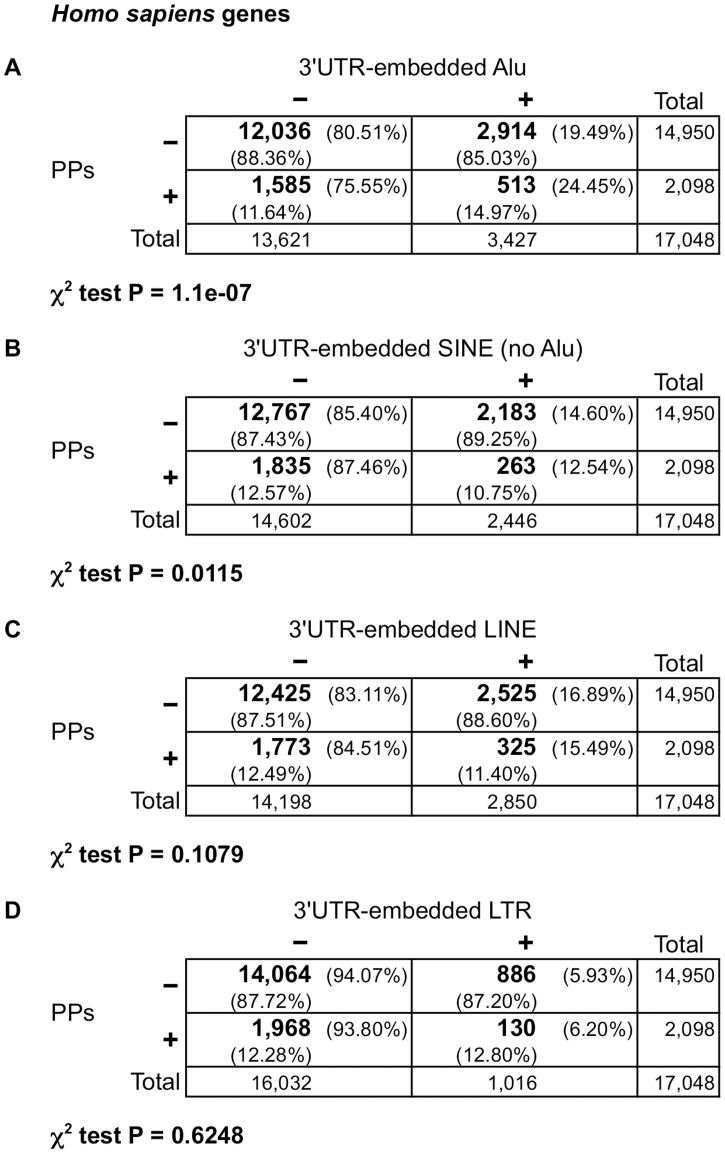
Contingence tables showing overrepresentation of Alu elements, but not of other retrotransposons, inside 3’UTRs of human PP parent genes. Plus and minus signs above the tables indicate presence or absence, respectively, of Alus (**A**), other SINEs (**B**), LINEs (**C**), or LTRs (**D**) inside the 3’UTR(s) of a gene. Plus and minus signs on the left mean presence or absence, respectively, of PPs generated from a gene. Numbers in bold are gene counts; total number of genes are also displayed in the right column and the bottom row for each table. Percentages with respect to each total are also shown. P-values of the χ^2^ test are indicated below each corresponding table.

As the reverse transcription of an mRNA starts from its 3’-end, we postulated that repeat elements present in 3’UTRs could affect the genesis of retrocopies. To study the putative effect of the presence of 3’UTR-embedded retrotransposons, especially Alu elements, on the existence of processed pseudogenes, we decided to cross-compare available human genome data related to these aspects. We downloaded human genes from Ensembl release 71 [57] and annotated those that have PPs using data from pseudogene.org [58]. Initial annotation of Alus and other SINEs embedded inside 3’UTRs was obtained from Transpogene [59]. This information was completed with Alus and other retrotransposons (other SINEs, LINEs, and long terminal repeats, LTRs) annotated in the UCSC Genome Browser database mapping to 3’UTRs as defined in Ensembl release 71 and/or RefSeq (hg19) [57, 60, 61]. Genes that have only monoexonic transcripts or do not code for a protein were discarded (see Methods for more details). Genes with only monoexonic transcripts were rejected because it is difficult to differentiate if they are PP parent genes or retrocopies. The result of this selection was a list of 17,048 human genes, among them 2,098 (12.31%) having generated PPs (Fig 1 and S1 Table).

We calculated that 3,427 (20.10%) human genes have Alu elements inside the 3’UTR of one or more of their transcripts (Fig 1A and S1 Table). Importantly, using a contingence table comparing the presence of 3’UTR-embedded Alus with the existence of PPs, we observed a significant overrepresentation of the presence of Alu elements in the 3’UTR(s) in those genes with PPs assigned (Fig 1A). 24.45% of genes with PPs have Alus in their 3’UTRs, comparing with 19.49% of genes without PPs (P < 10^−6^, χ^2^ test).

We also examined if the orientation of the Alus in 3’UTR could be related with the existence of PPs. To this end, we classified the genes that have Alus in their 3’UTRs in three groups: *sense* (all the 3’UTR-embedded Alu elements have the same sense that the containing gene), *antisense* (all the 3’UTR Alus are in the sense opposite to the gene), and *mix* (3’UTR Alus in both senses). As shown in S1 Fig, in the three groups there are genes with PPs, though there is a significant higher percentage in the *antisense* and *mix* groups (15.57% and 18.45%, respectively) comparing with the *sense* group (12.84%; P = 0.003, χ^2^ test). These results suggest that, apart of their location inside 3’UTRs, the relative orientation of Alu elements could contribute in some way to the genesis of PPs.

In contrast with the Alu elements, we found that the presence of other SINEs in the 3’UTR(s) is underrepresented in human PP parent genes (Fig 1B; P < 0.02, χ^2^ test); 12.54% of genes with PPs have non-Alu SINEs in their 3'UTR, compared with 14.60% of genes without PPs. Additionally, when we examined LINE and LTR retrotransposons inside 3’UTRs, mostly as fragments, no significant relationship with the presence of PPs was found (P > 0.1, χ^2^ test), as shown in Fig 1C (for LINEs) and Fig 1D (for LTRs).

As a further control, we examined whether the presence of Alus inside other regions of human genes could have a potential connection with the genesis of PPs. We explored this issue by analyzing 5’UTRs and intronic regions (see Methods for more details). As indicated by the corresponding contingence table, no association was found between Alus inside intronic regions and PP existence (S2 Fig; P > 0.9, χ^2^ test). In the case of 5’UTRs, however, a significant higher percentage of presence of Alus was found for genes with PPs (S2 Fig; P = 0.0116, χ^2^ test). It must be noted, though, that the number of genes that contain Alu elements in their 5’UTRs is relatively small, 354 genes (2.08%) of which only 59 have generated PPs, indicating that the putative contribution of 5’UTR-embedded Alus to the generation of PPs, if it exists, should be very weak.

Altogether, our results display an overrepresentation of Alu elements in the 3’UTRs of genes that produced PPs and suggest that this kind of SINEs contributes to the genesis of PPs. We speculate that, by binding to SRP9/14, the presence of these repeat structures inside the 3’UTR facilitates the way to the ribosomes in order to hijack the L1 machinery necessary to retrotranspose.

### Mouse and rat genomes also show overrepresentation of the presence of 3’UTR-embedded Alu-like elements in genes parent of processed pseudogenes

**Fig 2:**
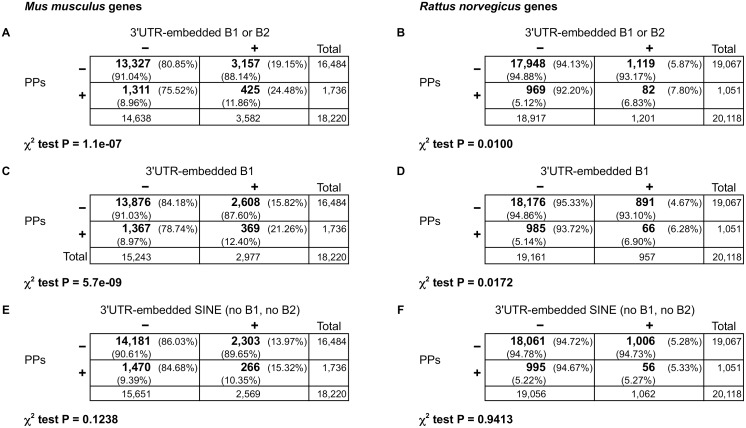
Contingence tables showing overrepresentation of Alu-like elements, but not of other SINEs, inside 3’UTRs of mouse and rat PP parent genes. Plus and minus signs above the tables indicate presence or absence, respectively, of B1 or B2 elements (**A**, **B**), B1 elements alone (**C**, **D**), or other SINEs (**E**, **F**) inside the 3’UTR(s) of a gene. Plus and minus signs on the left mean presence or absence, respectively, of PPs generated from a gene. Numbers in bold are gene counts; total number of genes are also displayed in the right column and the bottom row for each table. Percentages with respect to each total are also shown. P-values of the χ^2^ test are indicated below each corresponding table.

In order to determine whether the presence of Alu-like elements in the 3’UTRs of rodents show a similar relationship with the existence of processed pseudogenes, we extended our analysis to mouse and rat genomes. Genes were downloaded from Ensembl release 71 and annotated using pseudogene.org data [57, 58]. We also added information of SINEs (including B1 and B2 elements) annotated in the UCSC Genome Browser database that map to 3’UTRs as defined in Ensembl release 71 and/or RefSeq (mm10 and rn5 for mouse and rat, respectively) [57, 60, 61]. As in the case of human genes, those genes that have only monoexonic transcripts or do not code for a protein were discarded (more details are explained in Methods). 18,220 mouse genes and 20,118 rat genes were obtained (Fig 2, S2 and S3 Tables). 1,736 mouse genes (9.53%) and 1,051 rat genes (5.22%) are parent of PPs. Whereas 3,582 mouse genes (19.66%) have 3’UTR-embedded B1 or B2 elements, only 1,201 rat genes (5.97%) have these SINEs inside their 3’UTRs.

In the case of mouse, contingence tables showed a significant overrepresentation of the presence of B1 or B2 elements inside the 3’UTRs in genes that generated PPs: 24.48% of genes with PPs comparing with 19.15% of genes without PPs (Fig 2A; P < 10^−6^, χ^2^ test). Lower percentages but with a more significant difference were obtained considering only the presence of B1 in 3’UTRs: 21.26% comparing with 15.82% of genes without PPs (Fig 2C; P < 10^−8^, χ^2^ test). Contingence tables of rat data also revealed an overrepresentation of the presence of B1 (or the presence of B1 or B2) in the 3’UTRs of genes parent of PPs (Fig 2B and 2D; P < 0.02, χ^2^ test). However, compared with the mouse genes, the percentages of rat genes with 3'UTR-embedded B1s/B2s are considerably lower, less than 8% (Fig 2B and 2D); for instance, 7.80% of genes with PPs have B1 or B2 elements in their 3'UTR, compared with 5.87% of genes without PPs (Fig 2B). The P-values of the χ^2^ test in this case were also comparatively less significant. These differences between rat and mouse genes could be related to the poorer quality of rat 3'UTR annotations as suggests the higher abundance of genes without annotated 3’UTR in the rat dataset (5,925 genes, 29.45%; S3 Table) comparing with the mouse dataset (611 genes, 3.35%; S2 Table).

As SINE elements can appear or disappear from a 3’UTR of a gene in one species by insertion/deletion or by redefinition of the transcript end, we repeated the analysis combining mouse and rat data together, comparing the presence of 3’UTR-embedded B1/B2 elements and the generation of PPs in whatever of the two species. We obtained a list of 14,664 mouse and rat matching genes that show a clear overrepresentation of the existence of B1 (or the existence of B1 or B2) in the 3’UTRs of PP parent genes with higher percentages than considering only one species (S3 Fig). For instance, 28.39% of mouse/rat genes that generate PPs have B1 or B2 elements in their 3’UTRs, comparing with 21.32% of genes without PPs (P < 10^−9^, χ^2^ test).

In these analyses we included B2 elements, tRNA-derived SINEs without SRP9/14 binding capability, because it has been demonstrated that they also use L1 proteins to retrotranspose [10]. However, whereas the overrepresentation of 3’UTR-embedded B1s of PP parent genes is maintained when we considered 3’UTRs that present only B1 elements (no B2s or other SINEs), no over or underrepresentation was observed when 3’UTRs that present only B2 elements were considered (S4 Fig). Thus, the contribution of B2 elements to the genesis of PPs was not proved. Importantly, we also found that the presence of other SINEs (different from B1 and B2) in the 3’UTRs does not show a significant difference in mouse and rat PP parent genes (Fig 2E and 2F; P > 0.1, χ^2^ test).

Finally, we tested the link between PP genesis and the presence of B1 elements inside introns and 5’UTRs of mouse genes (see Methods for more details). No connection was found between B1s inside intron regions and PP existence (S5 Fig; P > 0.4, χ^2^ test). In the case of 5’UTRs, only 138 genes (0.76%) have B1 elements in these regions and a higher percentage was found for these genes with PPs but with a p-value in the limit of significance (S5 Fig; P = 0.0461, χ^2^ test). Therefore, altogether, our results show an overrepresentation of B1s in the 3’UTRs of genes that produced PPs in mouse and rat, suggesting that the contribution of Alu-like SINEs to the genesis of PPs is also present in rodent species.

### The overrepresentation of 3’UTR-embedded Alu elements in processed pseudogene parent genes is independent of transcript length and GC-content

**Fig 3:**
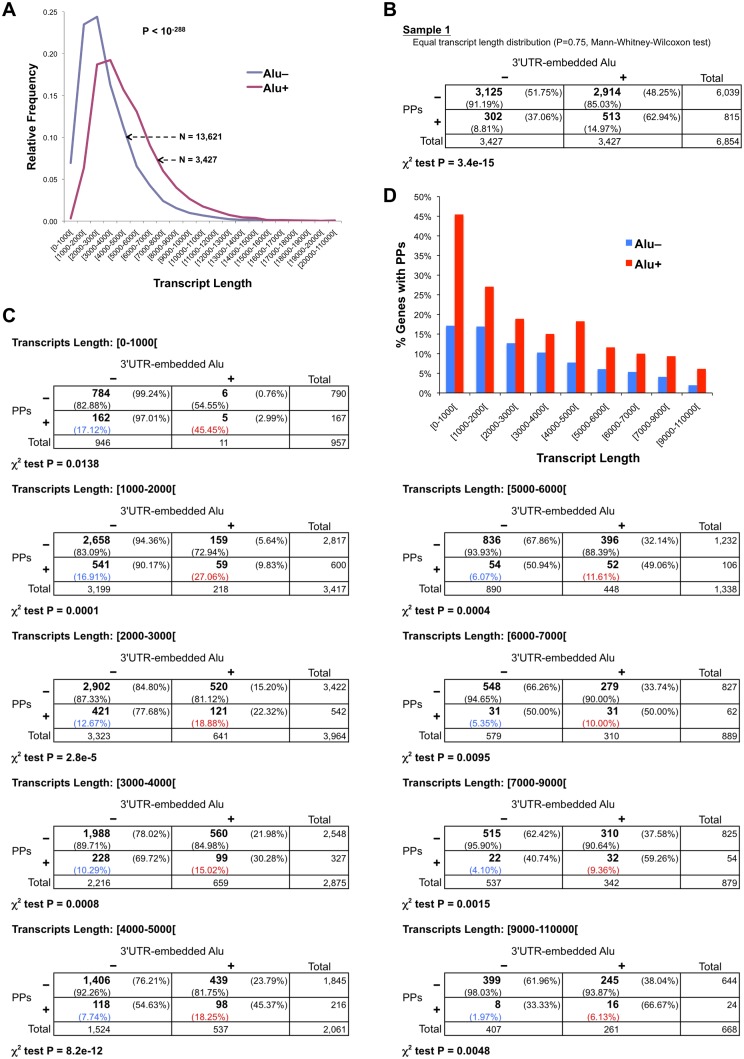
The overrepresentation of 3’UTR-embedded Alu elements in PP parent genes is independent of transcript length. (**A**) Transcript length distribution of genes with and without Alus in their 3’UTRs (Alu+ and Alu–). The P-value of the Mann-Whitney-Wilcoxon test comparing Alu+ and Alu− distributions and the number of genes (N) in each set are also indicated. (**B**) Sampling analysis to separate the possible effect of the transcript length (see Methods for details). Ten samples were generated. For each sample, Mann-Whitney-Wilcoxon (MWW) test proved that both gene sets (Alu+ and sampled Alu–) have a similar transcript length distribution and a contingence table showed overrepresentation of 3’UTR-embedded Alu elements in PP parent genes (χ^2^ tested). Here only the contingence table of the first sample is shown; see S7 Fig for the rest of the samples. (**C**) Contingence tables showing overrepresentation of Alu presence inside 3’UTRs of PP parent genes for each of the sets of genes grouped by their maximum transcript length (nine bins). (**D)** Percentage of genes that have PPs among 18 sets of genes grouped by their transcript length (the nine bins defined in **C**) and the presence or absence of 3’UTR-embedded Alu repeats (Alu+ and Alu–). The percentage values represented in **D** are also display in the contingence tables of **C** shaded in blue (Alu–) and red (Alu+). In **B** and **C**, plus and minus signs above the tables indicate presence or absence, respectively, of Alus inside the 3’UTR(s) of a gene. Plus and minus signs on the left of the tables mean presence or absence, respectively, of PPs generated from a gene. Numbers in bold are gene counts; total number of genes are also displayed in the right column and the bottom row of each table. Percentages with respect to each total are also shown. P-values of the χ^2^ test are indicated below the corresponding table.

We considered the possibility that differences in transcript length could explain the 3’UTR-embedded Alu overrepresentation in PP source genes, as an indirect effect. The reasoning of this consideration is based on the supposition that PP prediction algorithms are better detecting PPs generated from longer transcripts than from shorter ones. Then, the increase in transcript length as a result of Alu insertions could favor the detection of PP produced from transcripts with 3’UTR-embedded Alus, generating a false association between PP existence and Alu presence in 3’UTRs. To investigate this aspect, we calculated the maximum transcript length for each human gene (S1 Table; see Methods for details). Then we divided the genes in two sets: those that have Alu elements in 3’UTRs (Alu+; 3,427 genes) and those that do not have them (Alu−; 13,621 genes). In each set we grouped the genes by their maximum transcript length (1000 bp bins) and plotted their relative frequency in the corresponding set. As shown in Fig 3A, there is a clear difference between both distributions (P < 10^−288^, Mann-Whitney-Wilcoxon test), with Alu+ genes having longer transcripts than Alu− genes (mean of 4,888.97 bp and 3,381.58 bp, respectively). Thus, with the intention to remove the possible effect of the transcript length differences observed between Alu+ and Alu− genes, we conducted a sampling analysis. Briefly, we grouped the Alu+ and Alu− genes into nine bins based on their maximum transcript length. Next, we applied random sampling in each bin to generate a sample of Alu− genes with a similar distribution to the Alu+ gene set and the same number of genes (3,427 genes; S6 Fig; see Methods for more details). We generated ten samples using this method and all these samples confirmed the overrepresentation of the 3’UTR-embedded Alu elements in parent genes of processed pseudogenes (S7 Fig). The sample 1 is displayed in Fig 3B as an example, showing that 62.94% of genes with PPs have Alus in their 3’UTRs, comparing with 48.25% of genes without PPs (P < 10^−14^, χ^2^ test); Mann-Whitney-Wilcoxon test confirms that there are not significant differences in the transcript length distribution between the sampled Alu− gene set and the Alu+ gene set (P = 0.75). Moreover, the effect of the transcript length was also discarded using a different approach consisting in calculating the contingence table of PP existence and 3’UTR-embedded Alu presence for each of the nine bins of maximum transcript length. Fig 3C displays the nine resulting contingence tables; all of them indicate overrepresentation of Alu presence in the 3’UTRs of genes that produced PPs (P < 0.02, χ^2^ test).

Additionally, to better appreciate these results, we plotted the percentage of genes that have PPs for the nine bins of transcript length (the same bins defined in the previous analysis), distinguishing Alu+ and Alu− genes (Fig 3D). The percentage values plotted in Fig 3D appear shaded in blue (Alu–) and red (Alu+) inside the contingence tables of Fig 3C. Fig 3D shows that there is a decline in the percentage of PP source genes with the increase of transcript length, in agreement with another study that found a negative correlation between mRNA length and the number of pseudogenes [62]. Besides, the most interesting observation is that Alu+ genes show a higher percentage of PP parent genes for each bin, a significant difference as the contingence table analysis proved (Fig 3C; χ^2^ test). Thus, Fig 3D illustrates plainly that the relationship existing between the presence of 3’UTR-embedded Alu elements and processed pseudogenes does not depend of the transcript length.

**Fig 4:**
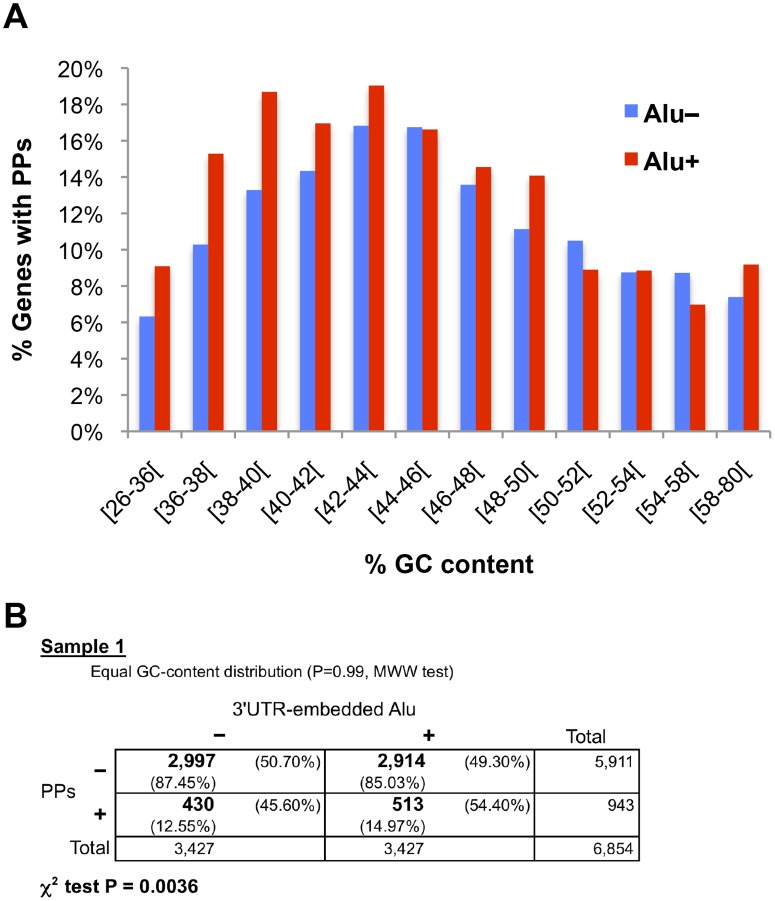
The overrepresentation of 3’UTR-embedded Alu elements in PP parent genes is independent of GC-content. (A) Percentage of genes that have PPs among 24 sets of genes grouped by their GC-content (12 bins) and the presence or absence of 3’UTR-embedded Alu repeats (Alu+ and Alu−). (B) Sampling analysis to separate the GC-content possible effect (see Methods for details). Ten samples were generated. For each sample, Mann-Whitney-Wilcoxon (MWW) test proved that both gene sets (Alu+ and sampled Alu−) have a similar GC-content distribution and a contingence table showed overrepresentation of 3’UTR-embedded Alu elements in genes with PPs (χ^2^ tested). Only the contingence table of the first sample is shown here; see S8 Fig for the rest of the samples. Plus and minus signs above the table indicate presence or absence, respectively, of Alus inside the 3’UTR(s) of a gene. Plus and minus signs on the left of the table mean presence or absence, respectively, of PPs generated from a gene. Numbers in bold are gene counts; total number of genes are also displayed in the right column and the bottom row of the table. Percentages with respect to each total are also shown. P-value of the χ^2^ test is also indicated.

Another factor that could be influencing the link between 3’UTR Alus and PP existence is the gene base composition. To assess this possibility we downloaded the percentage of GC (GC-content) for each gene from Ensembl using Biomart and added this information to the human dataset (S1 Table). Genes were grouped by GC-content in 12 bins (see Methods for details) and the percentages of Alu+ and Alu− genes that have PPs were plotted for each bin. As illustrated in Fig 4A, some changes are observed along the GC-content bins, but without a clear tendency in the differences between Alu+ and Alu− genes. To discard GC-content as a determinant factor of these differences, we used a sampling analysis similar to the one employed for the transcript length examination. We applied random sampling in each bin to obtain a sample of Alu− genes with the same cardinality as the Alu+ gene set (3,427 genes) and a similar distribution (see Methods for more details). As shown in S8 Fig, the ten samples generated using this method corroborated independently of the GC-content the overrepresentation of the 3’UTR-embedded Alu elements in PP source genes. For example, in the contingence table of sample 1 (also displayed in Fig 4B), 54.40% of genes with PPs have Alus in their 3’UTRs, comparing with 49.30% of genes without PPs (P < 0.004, χ^2^ test). Thus, taken as a whole, the overrepresentation of 3’UTR-embedded Alu elements in PP parent genes is not associated with GC-content.

### Lowly expressed genes that are source of processed pseudogenes show a higher tendency to 3’UTR-embedded Alu occurrence

Most genes that generated multiple PPs are highly expressed housekeeping genes [63]. On the other hand, some authors have suggested that 3’UTR retrotransposon insertions (including Alu repeats) reduce mRNA expression [64]. Thus, taken together these observations, one would expect that Alu elements inside 3’UTRs would reduce gene expression and, as a result, decrease the emergence of new PPs, being therefore in disagreement with our hypothesis that 3’UTR-embedded Alu elements contribute to the genesis of PPs. For this reason, we aimed to clarify the relationship between gene expression level and PP existence and how this relationship could be affected by the presence of Alus in 3’UTRs.

**Fig 5:**
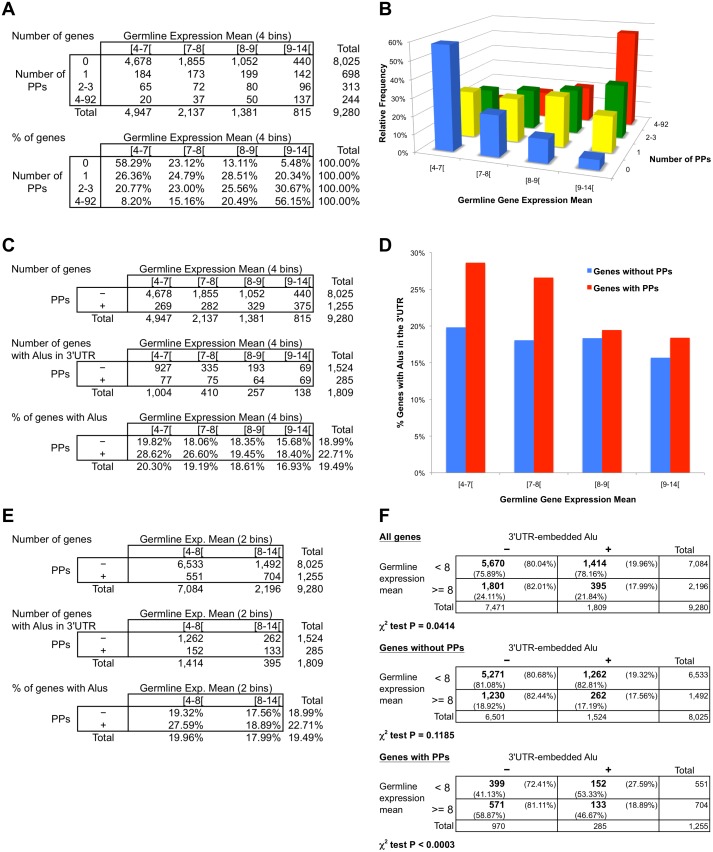
Gene expression and overrepresentation of 3’UTR-embedded Alu elements in PP parent genes. (**A**) The top table shows number of genes grouped by their germline gene expression mean and their number of generated PPs (4x4 groups); total numbers of genes are also displayed. The bottom table shows the corresponding percentage of genes in each row (groups by number of PPs) of the top table. (**B**) Bar graph representing the data from the bottom table of *A*. (**C**) Counts of genes grouped by their germline gene expression mean (4 bins) and presence or absence of generated PPs (+ and–, respectively). Total numbers of genes are also indicated. The top table displays the overall dataset; the middle table shows only genes with 3’UTR-embedded Alus; the bottom table presents the percentages of genes with these Alus. (**D**) Bar graph representing the data from the bottom table of **C**. (**E**) Same tables as in **C** but grouping genes into 2 bins of germline gene expression mean. (**F**) Contingence tables testing overrepresentation of Alu elements inside 3’UTRs of lowly expressed genes (germline gene expression mean lower than 8) respect to highly expressed genes (germline gene expression mean higher or equal to 8). The top table displays the overall gene set; the middle table shows only the genes without associated PPs; the bottom table presents only the genes with PPs. Plus and minus signs above the tables indicate presence or absence, respectively, of Alus inside the 3’UTR(s) of a gene. Numbers in bold are gene counts; total number of genes are also displayed in the right column and the bottom row for each table. Percentages with respect to each total are also shown. P-values of the χ^2^ test are indicated below each corresponding table.

With this purpose in mind, we downloaded data from a human gene expression study [65], estimated for each gene its gene expression mean in germline tissues, and combined these estimations with our PP parenthood and 3’UTR-embedded Alu data (see Methods for details). As changes in the genome are only transmitted to subsequent generations and fixed in the genome if they occur in germline cells, our interest was focused on germline gene expression. The resulting dataset has 9,280 genes (Fig 5A top table and S1 Table). We then divided the gene set in 4x4 bins based on their germline gene expression mean and the number of PPs they have. We calculated the number of genes in each bin (Fig 5A top table), the percentage of genes for each gene expression category respect to the total of each PP-counting group (Fig 5A bottom table), and represented these percentages in a column graph (Fig 5B). Most of the genes without PPs are lowly expressed genes (58.29%). By contrast, most genes with 4 or more PPs are highly expressed genes (56.15%). Thus, as expected, there is a clear positive correlation between germline gene expression and PP existence.

Our analysis also showed that the presence of Alu elements in 3’UTRs is higher in lower expressed genes. Fig 5C displays the number of total genes (top table), the number of genes with Alus in 3’UTR (middle table), and the percentage of genes with 3’UTR-embedded Alus (bottom table) in each gene expression group (4 bins) separating genes with PPs (+) from genes without PPs (–). These percentages are also graphically shown in Fig 5D, which helps to visualize that the percentage of genes with 3’UTR-embedded Alus differs between genes with and without PPs, particularly in the two groups with lower gene expression (germline gene expression mean < 8). To better appreciate these differences, Fig 5E shows the same information than in Fig 5C but grouping by germline gene expression in only 2 categories (< 8 or > = 8). In those genes with lower expression there is a clear contrast in the percentage of them that have 3’UTR-embedded Alus between PPs+ and PPs–(27.59% compared to 19.32%, respectively; Fig 5E bottom table). Fig 5E (bottom table) also indicates that 19.96% of genes with expression mean lower than 8 have 3’UTR-embedded Alus, whereas 17.99% of genes with higher expression have Alus inside their 3’UTRs. However, as shown in Fig 5F, this variation has a low significance (P = 0.04, χ^2^ test; Fig 5F top contingence table) and this significance disappears when only genes without PPs are considered (P = 0.1185, χ^2^ test; Fig 5F middle contingence table). In marked contrast, genes that generated one or more PPs showed a clear difference (P < 0.0003, χ^2^ test; Fig 5F bottom contingence table) between those genes with expression < 8 (27.59% of genes have 3’UTR-embedded Alus) and those with expression > = 8 (18.89% of Alu presence in 3’UTR). Thus, the differences in the existence of Alu elements inside 3’UTRs between lowly expressed and highly expressed genes are concentrated in those genes that are source of processed pseudogenes.

**Fig 6:**
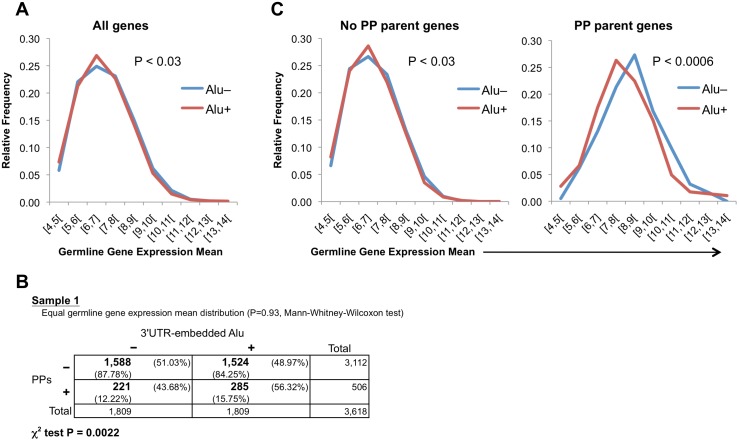
The 3’UTR-embedded Alu overrepresentation in PP parent genes is not a by-product of germline expression differences between Alu+ and Alu− genes. (**A**) Germline gene expression mean distribution of genes with and without Alus in their 3’UTRs (Alu+ and Alu–). The graph shows the overall gene set. The P-value of the Mann-Whitney-Wilcoxon test comparing Alu+ and Alu− distributions is also indicated. (**B**) Sampling analysis to separate the possible effect of the germline gene expression level (see Methods for details). Ten samples were generated. For each sample, Mann-Whitney-Wilcoxon (MWW) test proved that both gene sets (Alu+ and sampled Alu–) have a similar germline gene expression mean distribution and a contingence table showed overrepresentation of 3’UTR-embedded Alu elements in PP parent genes (χ^2^ tested). Here only the contingence table of the first sample is shown; see S9 Fig for the rest of the samples. Plus and minus signs above the table indicate presence or absence, respectively, of Alus inside the 3’UTR(s) of a gene. Plus and minus signs on the left of the table mean presence or absence, respectively, of PPs generated from a gene. Numbers in bold are gene counts; total number of genes are also displayed in the right column and the bottom row of the table. Percentages with respect to each total are also shown. P-values of the χ^2^ test are indicated below the table. (**C**) Germline gene expression mean distribution of genes with and without Alus in their 3’UTRs (Alu+ and Alu–). The left graph displays only the genes without associated PPs. The right graph presents only the genes with PPs. The P-values of the Mann-Whitney-Wilcoxon test comparing Alu+ and Alu− distributions are also indicated.

We also observed that, despite germline expression level distributions of Alu+ and Alu− genes are apparently very similar (Fig 6A), there is actually a significant difference between them (P < 0.03, Mann-Whitney-Wilcoxon test). Concerned with the possibility that this difference could in part explain the 3’UTR-embedded Alu overrepresentation in PP parent genes as a by-product, we applied a sampling analysis similar to the one used for the transcript length effect evaluation. Briefly, we grouped the Alu+ and Alu− genes into seven bins based on germline gene expression mean. Then we used random sampling in each bin to generate a sample of Alu− genes with a similar distribution to the Alu+ gene set and the same number of genes (1,809 genes; see Methods for more details). We created ten samples using this method and all of them confirmed the overrepresentation of the 3’UTR-embedded Alu elements in PP source genes (S9 Fig). The sample 1 is displayed in Fig 6B as an example, showing that 56.32% of genes with PPs have Alus in their 3’UTRs, comparing with 48.97% of genes without PPs (P < 0.003, χ^2^ test); Mann-Whitney-Wilcoxon test confirms that there are not significant differences in the germline expression level distribution between the sampled Alu− gene set and the Alu+ gene set (P = 0.93). Interestingly, whereas germline gene expression mean distributions of Alu+ and Alu− genes without PPs showed a difference similar to the complete gene dataset (P < 0.03, Mann-Whitney-Wilcoxon test; Fig 6C left graph), PP parent genes showed a stronger germline expression level difference between Alu+ and Alu− genes (P < 0.0006, Mann-Whitney-Wilcoxon test; Fig 6C right graph) with a clear tendency of Alu+ to lower gene expression level. This observation reinforces the conclusion that the differences between lowly expressed and highly expressed genes are centered in the PP parent genes. Our results may indicate that, in order to generate retropseudogenes, 3’UTR-embedded Alu elements are especially beneficial in lowly expressed genes. A plausible interpretation is that highly expressed genes have an elevated probability to hijack the ORF2 protein of L1 without need of Alu help, while the presence of 3’UTR Alus with its binding to SRP9/14 facilitates considerably ORF2p hijacking in lowly expressed genes.

### Presence of Alu elements in the 3’UTR of primate genes captured by herpesviruses

We then speculated that the same L1 machinery may also be used by large DNA viruses to capture genes from their hosts. In order to study the genesis of intronless viral genes homologous of host genes, we selected the large and highly species specific family of herpesviruses. We performed several NCBI BLAST searches of all the annotated protein sequences of herpesviruses that infect primates against the complete dataset of primate proteins using DELTA-BLAST [66]. Although numerous genes of large DNA viruses show homology to host genes, high divergence between the viral and host gene sequences makes almost always difficult to prove their common origin. Thus, the identification of the exact host parent gene of a viral homolog is remarkably complex, mainly because of the high evolutionary rates of viral genes [67]. For these reasons, our analysis of viral homologs was concentrated on the herpesviral genes that i) code proteins of 100 or more amino acids and ii) exhibit a high percentage of amino acid identity, equal or more than 60% and covering at least half of the viral protein, respect to their host parent genes (see Methods for more details). Apart from intronless viral genes, ORFs with only one intron were also included in the search because the intron could be originated after the capture from the host gene. Using these criteria, the analyses yielded 20 viral genes homologous to nine host genes (Table 1). Cellular genes were: interleukin 10 (*IL10*), dihydrofolate reductase (*DHFR*), signaling lymphocytic activation molecule (SLAM) family member 6 (*SLAMF6*, *CD352*), interleukin 17A (*IL17A*), thymidylate synthetase (*TYMS*), the complement regulatory protein *CD59*, the C-type lectin domain family 2 (CLEC2) like gene *LOC101037697*, and two additional SLAM family members, lymphocyte antigen 9 (*LY9*, *CD229*) and *CD48*.

**Table 1:** Primate genes captured by herpesviruses with high amino acid identity between host and viral proteins.

Host Gene	Host Species^a^	Viral Gene	Viral Species	AA Identity^b^
*IL10*	*Macaca fascicularis*	*BCRF1*	Lymphocryptovirus Macaca	89.35%
	*Macaca mulatta*	*BCRF1*	Macacine Herpesvirus 4	88.17%
	*Homo sapiens*	*BCRF1*	Epstein-Barr Virus (EBV)	84.52%
	*Papio anubis*	*vIL10*	Papiine Herpesvirus 1	81.87%
*DHFR*	*Saimiri sciureus*	*ORF2*	Herpesvirus Saimiri (HVS)	84.97%
	*Macaca nemestrina*	*ORF2*	Macaca nemestrina Rhadinovirus 2 (MneRV2)	61.96%
*SLAMF6*	*Saimiri sciureus*	*S1*	Squirrel Monkey Cytomegalovirus (SMCMV)	79.51%
*IL17A*	*Saimiri sciureus*	*ORF13*	Herpesvirus Saimiri (HVS)	74.83%
*TYMS*	*Erythrocebus patas*	*ORF13*	Simian Varicella Virus (SVV)	73.90%
	*Ateles spp*.	*ORF70*	Ateline Herpesvirus 3 (AtHV3)	71.72%
	*Saimiri sciureus*	*ORF70*	Herpesvirus Saimiri (HVS)	70.75%
	*Homo sapiens*	*ORF13*	Varicella Zoster Virus (VZV)	69.23%
	*Homo sapiens*	*ORF70*	Kaposi’s Sarcoma-associated Herpesvirus (KSHV)	69.01%
	*Macaca mulatta*	*ORF70*	Rhesus Monkey Rhadinovirus (RRV)	69.01%
	*Macaca nemestrina*	*ORF70*	Macaca nemestrina Rhadinovirus 2 (MneRV2)	67.73%
	*Macaca nemestrina*	*ORF70*	Retroperitoneal Fibromatosis-associated Herpesvirus (RFHV)	67.31%
*CD59*	*Saimiri sciureus*	*ORF15*	Herpesvirus Saimiri (HVS)	70.25%
*LOC101037697*^c^	*Saimiri sciureus*	*S28*	Squirrel Monkey Cytomegalovirus (SMCMV)	64.42%
*LY9*	*Aotus trivirgatus*	*A33*	Owl Monkey Cytomegalovirus (OMCMV)	63.64%
*CD48*	*Aotus trivirgatus*	*A43*	Owl Monkey Cytomegalovirus (OMCMV)	62.28%

^a^When the amino acid sequence of the host species is not known, the amino acid sequence of the nearest species was used. This was the case for *Saimiri sciureus*, *Aotus trivirgatus*, *Erythrocebus patas*, and *Ateles spp*. where *Saimiri boliviensis*, *Aotus nancymaae*, *Macaca mulatta*, and *Aotus nancymaae*, respectively, were used.

^b^Amino acid identity between the viral protein and the host cellular protein of the indicated nearest species.

^c^CLEC2-like gene.

Table 1 includes additional information on the viral genes obtained from the search, such as viral species containing them and percentage of amino acid identities of the encoded proteins with respect to the corresponding cellular proteins. Four viral homologs of *IL10*, named *BCRF1* or *vIL10*, were identified in four gammaherpesviruses of the same group, including the well-known functional *IL10* homolog of the human Epstein-Barr virus [68, 69]. Amino acid identities of the viral products ranged from 81.87% to 89.35% respect to their host homologs. Presumably, the origin of these *IL10* homologs was a unique capture event in the ancestor of these evolutionarily very close gammaherpesviruses. Interestingly, homologs of *IL10* not fulfilling the searching criteria we employed are also present in other herpesviruses and some poxviruses that infect different vertebrates, as horses or sheep, in a remarkable phenomenon of independent acquisition of a cellular gene by viruses [55, 70]. Two homologs of *DHFR* were also retrieved from the search in the herpesvirus saimiri (HVS), a herpesvirus that infects New World (NW) monkeys, and the Macaca nemestrina rhadinovirus 2 (MneRV2) with amino acid identities of 84.97% and 61.96%, respectively. As expected from a previous study [71], more divergent *DHFR* homologs appeared also in other gammaherpesviruses, with amino acid identities below the 60% threshold established in our analysis. With a high degree of conservation (near 80% of amino acid identity), we also identified a homolog of *SLAMF6*, named *S1*, inside the squirrel monkey cytomegalovirus (SMCMV), another herpesvirus that infects NW monkeys. We have recently characterized this viral gene and reported that its protein product, S1, maintains SLAMF6 ligand capacities [72]. *ORF13*, a homolog of *IL17A* [73], and *ORF15*, a homolog of *CD59* [74] appeared also in HVS, with amino acid identities of 74.83% and 70.25%, respectively. These two genes are not found in other herpesviruses and our phylogenetic analysis suggests recent captures of *IL17A* and *CD59* by HVS (S10 Fig). Homologs of *TYMS* were found in eight alpha and gammaherpesviruses, being the *ORF13* in the Simian varicella virus (SVV) the viral *TYMS* homolog with the highest conservation: 73.90% of amino acid identity respect to its host counterpart. Apart from these eight viral genes, there are *TYMS* homologs with higher divergence in most gammaherpesviruses and some alphaherpesviruses, including virus that infect horses, and therefore we postulate that *TYMS* viral capture was very ancient; this idea was also supported by our phylogenetic analyses (S11 Fig). The search also yielded open reading frame *S28* of SMCMV showing a 64.42% homology with a gene of *Saimiri boliviensis* annotated as *LOC101037697* in the NCBI Gene Database, orthologous to a human CLEC2D pseudogene annotated as *LOC374443*. Finally, *A33* and *A43*, whose protein products display 63.64% and 62.28% amino acid identities with LY9 and CD48, respectively, were identified in another herpesvirus that infects NW monkeys, the owl monkey cytomegalovirus (OMCMV). We have recently shown through phylogenetic analysis that *A33* and *A43* were acquired by retrotranscription at two different moments of the virus-host coevolution and that they conserve LY9 and CD48 ligand binding properties, respectively [72]. Additional homologs of CD48 with lower amino acid identities were also found in SMCMV and OMCMV [72].

**Fig 7:**
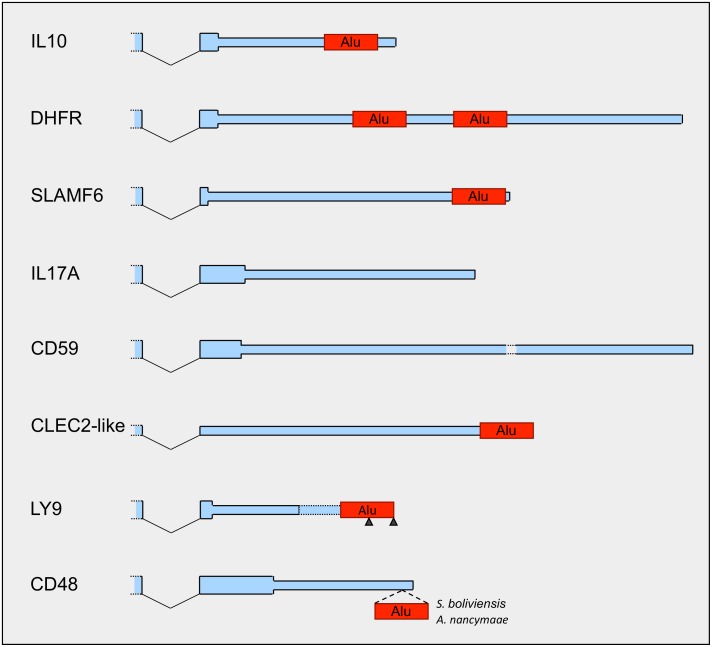
Primate genes captured by herpesviruses have Alu elements inside their 3’UTRs. Blue areas illustrate the last exon of the transcripts of the *IL10*, *DHFR*, *SLAMF6*, *IL17A*, *CD59*, CLEC2-like (*LOC101037697/LOC374443*), *LY9*, and *CD48* genes, where the narrower ending segment indicates the 3’UTR. The edge of the previous exon is also displayed (left open-ended rectangle). Exons are drawn to the same scale with respect to the human genome annotation, except for the CD59 3’UTR that was cut in the middle (void space with dotted lines) because it is very long. Black oblique lines represent splicing. For LY9, black triangles indicate alternative predicted polyadenylation sites and the dotted lines display a predicted 3’UTR addition. Red rectangles show the position of the Alu elements. IL17A and CD59 3’UTRs do not have Alu elements. In CD48, as indicated, the Alu element is only present in *S*. *boliviensis* and *A*. *nancymaae*.

After this searching process, we performed a close inspection of the 3’UTRs of these selected cellular genes, with a particular focus on the presence of Alu elements. Due to its very ancient viral capture, *TYMS* was discarded from the analysis, because it could have been incorporated to the ancestral herpesvirus using a different mechanism and/or the fingerprints of how it was captured could be lost. As illustrated in Fig 7, the analysis revealed that most of these host genes present one or two Alu elements inside their 3’UTRs. This is clear for *IL10*, *DHFR*, and *SLAMF6* where these 3’UTR-embedded Alus are present in human and the other primate genomes annotated in the UCSC Genome Browser database [61]. The human *IL17A* and *CD59* do not have Alu elements in its 3’UTR and there is not evidence of their existence inside the 3’UTR of these genes in *S*. *boliviensis*. However, as there are Alu elements in the intergenic region near after *IL17A* and *CD59*, we cannot discard a putative exonization of these Alus, being incorporated into alternative 3’UTRs, similarly to other recently described examples [75]. Alternatively, as we do not dispose of the genome sequence of *Saimiri sciureus*, the natural host of HVS, we cannot discard the existence of a new Alu inside the 3’UTRs of *IL17A* and *CD59* in this NW monkey species. With regard to the CLEC2-like gene, the human homolog *LOC374443*, despite being annotated as a pseudogene, has several expressed transcripts and one of these transcripts, CLEC2DandNPM1P16andNPM1P7.tAug10 annotated in AceView [76], ends with an Alu element that is also conserved in the genome of *S*. *boliviensis* (Fig 7). In the case of *LY9*, while the human *LY9* does not show an Alu element inside the annotated 3’UTRs, there are two alternative predicted polyadenylation sites, supported by two expressed sequence tags (ESTs) (GenBank IDs: CD639491 and DB333306), that extend the 3’UTR of the longest transcript and include half or the entire Alu annotated immediately 3’ downstream of *LY9* (Fig 7). This Alu is conserved in most primates and there is an EST of the NW monkey *Callithrix jacchus* (GenBank ID: HX541208) that also supports the predicted additional polyadenylation sites. Therefore, it is likely the existence in primates of LY9 transcripts with longer 3’UTR including this Alu element. Finally, Alu elements are absent from the 3’UTRs of the CD48 transcripts of all primates included in the UCSC Genome Browser database. However, the sequence alignment of the human CD48 isoform 2 transcript (RefSeq ID: NM_001256030) with the corresponding genomic sequences of the three NW monkeys currently sequenced, *C*. *jacchus*, *S*. *boliviensis*, and *Aotus nancymaae*, showed the existence of a new Alu in *S*. *boliviensis* and *A*. *nancymaae* inside the 3’UTR extrapolated from the human annotation (Fig 7 and S12 Fig). This new Alu was inserted inside an existing MIR probably after the split between the lineages of *Aotus/Saimiri spp*. and *Callithrix spp*. (S12 Fig). Interestingly, SMCMV and OMCMV naturally infect *S*. *sciureus* and *Aotus trivirgatus*, respectively, two NW monkey species evolutionarily closer to *S*. *boliviensis* and *A*. *nancymaae* than to *C*. *jacchus*. Taking into account that *CD48* homologs have been found only in these two NW monkey cytomegaloviruses, the appearance of this new Alu element may be related with the acquisition of the host *CD48* gene by these viruses.

Considering that 75% (6 out of 8) of genes that were captured by herpesviruses contain Alu elements in their 3’UTRs, exceeding substantially the 20.10% (3,427 out of 17,048 genes; Fig 1A) of mean of 3’UTR-embedded Alu presence in human genes, our results suggest that the existence of 3’UTR-embedded Alus could be related to the gene capturing process. Moreover, as our previous results indicate that the presence of Alus in 3’UTRs facilitates the genesis of processed pseudogenes, driven by the L1 encoded proteins, our findings support our hypothesis that it is the L1 machinery the cellular mechanism that herpesviruses use to incorporate host genes as new intronless viral genes.

## Discussion

Initially regarded as junk DNA, transposable elements are currently considered as intracellular parasites becoming domesticated during the course of evolution. Alus are not neutral parasites as Alu/Alu homologous recombination events and new Alu insertions may generate several diseases [2, 13, 77], but they are a source of genomic innovation contributing to genome plasticity [77–79]. Some biological functions have been postulated to explain the maintenance of Alu elements in primate genomes [80] and numerous occurrences of Alu exaptations (Alu exonization or generation of novel regulatory elements) have been reported [81–85]. Regarding Alu sequences embedded inside mRNAs, there is a tendency to accumulate Alu elements in 3’UTRs as compared to 5’UTRs [39]. In this connection, previous studies proposed a role of 3’UTR-embedded Alus in regulating mRNA stability. While Wilson et al. [40] demonstrated that Alus at 3’UTR increase mRNA half-life, An et al. [86] suggested that 3’UTR Alus could generate AU-rich elements that destabilize certain mRNAs. Other authors pointed to Alu RNAs embedded in 3’UTRs as microRNA targets that might influence gene expression [41]. However, it has been recently demonstrated that the potential microRNA targets within Alu sequences, in particular those in 3’UTRs, are largely non-functional and ignored by the microRNA machinery [87]. Other mechanisms of gene expression inhibition by 3’UTR inverted Alu repeats (two contiguous Alus in opposite sense) have been proposed, but they are controversial [88, 89]. Thus, whereas one out of every five human genes (3,427/17,048) has one or more Alu repeats in its 3'UTR(s) (Fig 1A), the functional significance of these elements remains elusive.

**Fig 8:**
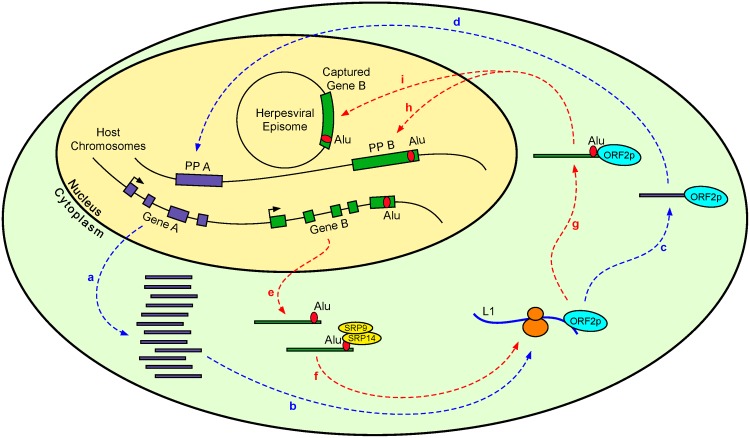
Schematic diagram illustrating the proposed hypothesis of the new role of 3’UTR-embedded Alus in the genesis of PPs and the herpesviral capture of host genes. Blue pathway: A highly expressed gene A produces a large amount of transcripts (**a**) and thus there is a high probability for one of these transcripts to come into contact with a ribosome that is translating an L1 RNA and bind the L1 ORF2p (**b**), steal it (**c**), and move back to the nucleus where the ORF2p is used to generate a new processed pseudogene of the gene A (PP A) (**d**). Red pathway: The few transcripts of a lowly expressed gene B (**e**) have, by contrast, a low probability to reach a ribosome that is translating an L1 RNA. However, the presence of an Alu element inside the 3’UTR of the gene B allows gene B transcripts to bind the abundant protein complex SRP9/14, promoting transcripts to move to the ribosomes and therefore increasing the likelihood to make contact with a ribosome that is translating an L1 RNA and bind the L1 ORF2p (**f**), steal it (**g**), and move to the nucleus where the ORF2p is used to generate a new processed pseudogene of the gene B (PP B) (**h**) or to insert a transcript retrocopy inside an existing herpesviral episome (the circled DNA of a herpesvirus) (**i**).

The results of the present study suggest a novel role for 3’UTR-embedded Alu repeats in the genesis of processed pseudogenes. We discovered overrepresentation of Alu elements in the 3’UTRs of genes source of processed pseudogenes, independently of their transcript length, GC-content, and gene expression level. Rodent Alu-like (B1) elements in 3’UTRs are also overrepresented in PP parent genes. The occurrence of this phenomenon in both primates and rodents denotes some functionality of these 3’UTR-embedded retroelements related to PPs. We postulate that the presence of SINEs of this class in a 3’UTR promotes the way to the ribosomes by binding to SRP9/14, increasing the likelihood of hijacking the L1 machinery to be retrotransposed (Fig 8). Our results also showed that the presence of Alus inside 3’UTRs is higher in lowly expressed genes and that this difference is concentrated in those genes parent of PPs, suggesting that 3’UTR-embedded Alus are especially useful in lowly expressed genes in order to generate retropseudogenes. We speculate that the presence of Alu elements in a 3’UTR elevates considerably the probability to hijack the ORF2 protein of L1 especially in those genes that generate very few transcripts (Fig 8).

L1 can function in *trans* to mobilize different kinds of cellular RNAs, including mRNAs that generate PPs, though at a much lower efficiency than in *cis* (with their own L1 mRNA) [26, 42, 90, 91]. This *cis*-preference is very effectively bypassed by Alu elements [9]. It has been suggested that this Alu retrotransposition efficiency is linked to the association between Alu RNA and SRP9/14 by directing them to ribosomes that are translating L1 transcripts, in close vicinity to the nascent L1 proteins [9]. This model is supported by the long-lasting conservation of the Alu secondary structure that binds the SRP9/14 heterodimer. It is probable that Alus inside mRNA 3’UTRs could also recognize the SRP9/14 proteins and increase considerably the retrotransposition of these mRNAs. Interestingly, Hasler et al. [39] indicated that SRP9/14 can bind to some Alu RNAs embedded in 3’UTRs. Moreover, SRP9/14 are present in a large excess over SRP in mammalian cells [28], available to be bound by free and 3’UTR-embedded Alu elements. Our results indicate that the orientation of the Alus inside 3’UTRs relative to the gene sense might also have an effect in the genesis of PPs. The presence of antisense Alus, which are generators of AU-rich elements, or inverted Alus could modify the localization of the transcript in the cytoplasm [86, 89], maybe increasing the probability of binding the SRP9/14 proteins.

Gene retrotransposition has long been considered a mechanism without functional significance. However, despite most processed pseudogenes have no function assigned, during last years several studies have shown that a considerable number of retrocopies have evolved into bona fide genes [92–97] and other retropseudogenes could play gene expression regulatory roles [98–100]. One of these PPs that are in fact bona fine genes is a retrocopy of *DHFR* [96], a parent gene that has also generated retrogenes in several gammaherpesviruses as mentioned previously and indicated in Table 1. Furthermore, a recent study identified in the human genome 25 “orphan” retrogenes that likely replaced their parent genes, which are pseudogenized or completely lost [101]. Thus, gene retrotransposition is regarded now as an important source of genetic innovation during mammalian evolution.

Besides advantageous genetic innovations, new processed pseudogenes could produce very detrimental effects. Recently, de Boer et al. published a case of chronic granulomatous disease affecting a man whose genome has a novel *TMF1* pseudogene inserted inside the first intron of the *CYBB* gene, causing aberrant *CYBB* mRNA splicing [102]. Interestingly, the TMF1 transcript that generated this pseudogene during early embryonic development was a non-annotated truncated transcript with a new 3’UTR that contains an Alu element. More available data about co-occurrence of PP polymorphisms and 3’UTR-embedded Alu polymorphisms would be very useful to further support our hypothesis of Alus inside 3’UTRs as facilitators of PP genesis.

Highly expressed housekeeping genes, such as those encoding ribosomal proteins and GAPDH, have generated a large number of PPs whereas most parent genes have just one or few PPs [46, 48]. This previous observation was confirmed by our results (Fig 5A and 5B). Several authors have opined that some kind of selection should exist to explain why some transcripts are retrotranscribed while the majority of them are not [43, 103]. One form of selection might be high expression of those transcripts in the germline. However, 55% of high expressed genes (440/815; Fig 5A top table) do not form PPs whereas there are lowly expressed genes with one or more PPs. Our study suggests that Alu repeats inside 3’UTRs facilitate retrotranposition of their containing genes, especially of those with low expression. Our sampling analysis indicates that the overrepresentation of the 3’UTR-embedded Alu elements in PP parent genes is not an effect of gene expression differences. Some authors have proposed that retrotransposons inside 3’UTRs (including Alus) reduce mRNA expression [64] and this could be an explanation for some of the differences we observed between the gene expression mean distributions of Alu+ and Alu− genes. However, we found that the differences in the existence of Alu elements inside 3’UTRs between lowly expressed and highly expressed genes are mostly concentrated in those genes that generated PPs. Interestingly, lowly expressed genes in general tend to express alternative polyadenylation isoforms with longer 3’UTRs than highly expressed genes [104]. In contrast with 3’UTR-embedded Alus, the weak association observed between 5’UTR Alus and PP existence could be explained by the high expression of the genes implicated, as 24 of the 59 genes with PPs and 5’UTR Alus (60% of the 40 genes with expression data) have expression higher than 8, while 19 genes (40%) have lower expression.

Retroviruses (exogenous and endogenous retroviruses) were initially postulated to mobilize Alu elements and generate processed pseudogenes [105]. However, several attempts to test this hypothesis were not successful [9, 42, 106]. Moreover, several studies have proved that the other possible source of reverse transcriptase, L1 ORF2p, can mobilize Alus, B1s, and B2s and generate PPs [26, 42, 107]. Thus, L1 ORF2p has been accepted as the mechanism to insert new copies of these elements into the genome.

We considered the possibility that a similar situation has also occurred in the effort to understand the mechanism of host gene capture by viral genomes. Large DNA viruses encode numerous genes homologous of cellular genes. Although direct recombination could explain the acquisition of some of them [108], this mechanism can hardly operate in host gene captures by viral genomes that replicate in the cytoplasm, not in close proximity to the cellular genome, as poxvirus genomes, for instance. Moreover, direct recombination is scarcely compatible with the fact that most viral homologs are intronless, which is better explained by retrotranscription of spliced cellular mRNAs. Therefore, in a similar way to the genesis of new Alu and PP copies, viral capture of host genes that produces new intronless homologous genes in herpesviruses and poxviruses has been classically explained by the presence of reverse transcriptase from retrovirus co-infecting the host cell or endogenous retroviruses [56, 70, 109]. However, this assumption has never been tested. Instead, the observation that herpesvirus homologs largely resemble processed pseudogenes [56] suggests that the L1 machinery, the mechanism that generates PPs, may also explain viral capture of host genes. The results of our analysis indicate that 75% of genes that were captured by herpesviruses contain Alu elements in their 3’UTRs. This exceeds considerably the mean of 3’UTR-embedded Alu presence in human genes, 20.10%. Although we did not found evidences of the presence of Alus inside the 3’UTR of two genes, *IL17A* and *CD59*, which were presumably incorporated recently to HVS, we cannot absolutely discard the presence of Alu elements in the 3’UTR of these host genes, as the genomic sequence of the HVS host, *S*. *sciureus*, has not been sequenced yet. Therefore, our results indicate that herpesviruses and possibly other large DNA viruses use the same host system that engenders processed pseudogenes, the L1 machinery aided by the presence of 3’UTR-embedded Alus, to incorporate host genes to the viral genome (Fig 8). Future advancing in complete genome sequencing of DNA viruses will make available more cases of captured host genes that should provide a more robust support to this hypothesis.

In order to generate PPs, the transcription of the source genes should coincide with the period when L1 ORF2p is available. Although L1 transcription may occur in a variety of cell types, only the results of retrotransposition in primordial germ cells, germline, or the early embryo can contribute to future generations. Apart from germline expression, L1 protein synthesis is elevated in early embryogenesis [6, 110–112]. Also accumulated evidences show L1-mediated retrotransposition activity, including PP genesis, in somatic cells [18, 103, 113]. Thus, if viral capture of host genes uses the same mechanism as PP genesis (the L1 machinery), it may occur either in germline or somatic tissues covering a broad cell spectrum. Gene hijacking by viruses may also occur in cancer cells, where L1 proteins could be very abundant [114]. We also speculate that the role of 3’UTR-embedded Alus facilitating the genesis of new retrocopies of lowly expressed genes may be important for some DNA viruses, as the incorporation to their genomes of lowly expressed cell-specific genes could be more useful for the virus that the capture of highly expressed housekeeping genes. In fact, most captured host genes appearing in Table 1 are lowly expressed in the majority of human tissues. Table 1 also shows that many of these genes were captured by herpesviruses that infect NW monkeys, which suggests a higher activity of LINE1s in this primate linage, in concordance with the large number of gene retrocopies observed in the marmoset (*Callithrix jacchus*) and squirrel monkey (*Saimiri boliviensis*) genomes [115, 116].

In addition to the limitation of the Pol III promoter mentioned in the introduction, Pol III-directed Alu transcription is inhibited by methylation [34], causing the low level of Alu expression. Therefore, embedding Alu elements inside 3’UTRs of Pol II transcribed genes seems a beneficial mechanism for those elements to spread across the genome when L1 machinery is available. Thus, presuming that the generation of processed pseudogenes as a source of genome novelty could be beneficial to species, both Alu elements and their hosts profit from Alu insertion inside 3’UTRs. Recently, Oliver and Greene postulated the TE-Thrust hypothesis that states that transposable elements (TEs) are powerful promoters of evolution [84, 117]. TEs can generate novelties in both an active mode (by retrotransposition, originating TE exaptations) and a passive mode (by DNA recombination resulting in genomic deletions, duplications, or rearrangements). Both modes are prone to TE sequence degeneration by accumulation of mutations. In this conceptual context, our hypothesis would add a new mode of TE-driven novelty generation that tends to maintain the structure and sequence of the involved TE, an Alu or Alu-like element, as its beneficial role depends on conserving its structure and particularly the SRP9/14 binding domains. This introduces a classical symbiotic situation, a mutualistic relationship, because the host, at the genome, cell, organism, or species level [118], would profit from a greater potential to generate processed pseudogenes and the Alu would maintain its sequence and structure and could also be retrotransposed along with its hosting gene.

## Conclusions

This study proposes a complete novel role for 3’UTR-embedded Alu elements as facilitators of the genesis of processed pseudogenes, especially in lowly expressed parent genes. This role is probably also attributable to the Alu-like B1 elements inside the 3’UTRs of rodent genomes as we observed a similar overrepresentation of these elements in mouse and rat genes source of processed pseudogenes. Additionally, we hypothesize that large DNA viruses exploit this L1-driven and Alu-aided retrocopy cellular mechanism in order to capture host genes for their own benefit. Future investigations should contribute to further support our hypothesis and to clarify the role of retrotransposons in the evolution of mammals and their viruses.

## Methods

### 3’UTR annotations

Human, mouse, and rat complete gene sets were downloaded from Ensembl release 71 [57]. 3’UTR genome annotations for those genes were collected from Ensembl release 71 and RefSeq (hg19, mm10, and rn5 for human, mouse, and rat genes, respectively) [57, 60]. Readthrough transcripts were filtered out. Genes annotated in chromosomes different from chr1-22, X, and Y were removed from the data sets. Genes without protein-coding transcripts or with only monoexonic transcripts were also discarded. The final data sets contain 17,048 human genes, 18,220 mouse genes, and 20,118 rat genes and they were included in the S1–S3 Tables. Ensembl gene IDs were used along our study to identify human, mouse, and rat genes. Biomart [119] was used to convert UCSC and RefSeq IDs to Ensembl IDs when necessary.

### Processed pseudogene annotations

Lists of processed pseudogenes were obtained from pseudogenes.org [58]; files Human71.txt, Mouse60.txt, and Rat50.txt were downloaded from this database and linked with the gene data sets using the Ensembl gene ID present in the *Parent Gene* field of these pseudogenes.org files. Only rows marked as “Processed” in the *Class* field were considered. The number of PPs of each parent gene was calculated and included in the S1–S3 Tables.

### Retrotransposon annotations

Initial annotation of SINEs (including Alus) embedded inside 3’UTRs of human genes was obtained from Transpogene [59]. This information was completed with SINE, LINE, and LTR retrotransposons annotated in the UCSC Genome Browser database mapping to 3’UTRs as defined in Ensembl release 71 and/or RefSeq (hg19) [57, 60, 61]. The annotation of SINEs (including B1 and B2 elements) embedded inside 3’UTRs of mouse and rat genes, as defined in Ensembl release 71 and/or RefSeq (mm10 or rn5), was also obtained from the UCSC Genome Browser database. The information about the existence of 3’UTR annotation and the presence of the different kind of retrotransposons (Alu elements, other SINEs, LINEs, and LTRs for human genes; B1 elements, B2 elements, and other SINEs for mouse and rat genes) was included in the S1–S3 Tables. Annotations of Alu and B1 elements from the UCSC Genome Browser database mapping to 5’UTRs and intronic regions as defined in Ensembl release 71 and/or RefSeq (hg19) were also added to the S1 and S2 Tables. In this study, for genes with three or more intronic regions, the last two were excluded from the Alu and B1 mapping because they usually overlap with 3’UTRs. Information about the orientation of the Alu elements inside the 3’UTRs of human genes was also added to the S1 Table, where genes with 3’UTR Alus were classified as *sense* (all the 3’UTR-embedded Alu elements have the same sense that the containing gene), *antisense* (all the 3’UTR Alus are in the sense opposite to the gene), and *mix* (3’UTR Alus in both senses).

### Estimation of GC-content and maximum transcript length of human genes

Percentages of GC for the human genes were downloaded from Ensembl release 71 using Biomart and included in the S1 Table. Lengths of all human transcripts annotated in Ensembl release 71 and RefSeq (hg19) were downloaded. The maximum transcript length for each human gene was calculated using Microsoft Excel and added to the S1 Table.

### Germline gene expression estimation

Human gene expression data was obtained from the study of McVicker and Green [65]. Germline tissue samples used in our study to estimate germline gene expression are listed in the S4 Table. Using the expression values from the file combined.rma.gene.expr.txt of the McVicker and Green’s study, the mean of gene expression level on the different germline samples (germline gene expression mean) was calculated for each gene using Microsoft Excel and added to the S1 Table.

### Statistical analysis

Column and line graphs, contingence tables, and χ^2^ tests were calculated using Microsoft Excel. Two-sample Mann-Whitney-Wilcoxon tests were performed in R 3.1.3 [120] by applying the wilcox.test function.

### Sampling analysis

Sampling analysis was performed to discard the possibility that the overrepresentation of 3’UTR-embedded Alus in PP source genes could be an indirect effect of transcript length, GC-content, or gene expression differences. Genes were grouped by maximum transcript length (nine bins), GC-content (twelve bins), or germline gene expression mean (seven bins). As the Alu− gene set contains in each bin higher number of genes than the Alu+ gene set, a simple random sampling was applied for each bin to generate a sample of Alu− genes with a similar distribution to the Alu+ genes. Random sampling was computed using an in-home Perl script that executes R 3.1.3 [120] to apply the srswor function (S1 File). The overrepresentation of the 3’UTR-embedded Alu elements in PP parent genes was tested in the ten samples (each one composed by the sampled Alu− genes and the original Alu+ genes) generated using this method.

### Identification of the host parent genes of herpesviral homologs

NCBI’s blast tools [66] were used to identify the host parent genes of the herpesviral homologous genes. All the sequences of herpesviral proteins with 100 or more amino acids encoded by genes with one or two exons were searched in the "Non-redundant protein sequences" database using DELTA-BLAST (limiting the search to primate proteins). The results with lower E-value corresponding to the host species (or the nearest species with known protein sequences) were selected. Complete host and viral amino acid sequences of the selected proteins were aligned using MAFFT online version 7 [121]. The S5 Table lists the GenBank IDs of the viral and primate protein sequences aligned. The resulting alignments were manually curated and the amino acid identity was calculated after removing positions with gaps (S2 File). Only host proteins with more than 60% of amino acid identity covering at least half of the viral protein were finally selected. The genes encoding these proteins were considered the host parent genes of the viral homologs.

### Alignment of primate CD48 3’UTR sequences

The sequence of CD48 isoform 2 transcript (RefSeq ID: NM_001256030) was used to map the corresponding homologous transcripts in the genomic sequences of *Callithrix jacchus* (GenBank ID: NC_013913), *Saimiri boliviensis* (GenBank ID: NW_003943698), and *Aotus nancymaae* (GenBank ID: NW_012166091) using the NCBI’s blastn [66]. The sequences of the last exon and the 3’UTR of the four species were aligned using MAFFT online version 7 [121]. Repeat elements in the 3’UTR sequences were predicted using CENSOR [122].

### Maximum Likelihood phylogenetic analysis

The S6 Table lists the GenBank and Ensembl IDs of IL17A, CD59, and TYMS transcript sequences used for the phylogenetic analysis. Herpesviral open reading frame sequences were extracted from the genomic sequences whose GenBank IDs are also displayed in the S6 Table. The DNA sequences were aligned using MAFFT online version 7 [121]. The evolutionary history was inferred by using the Maximum Likelihood method based on the Hasegawa-Kishino-Yano (HKY85) model [123], applying a bootstrap test of 1000 replicates [124]. The percentage of replicate trees in which the associated taxa clustered together in the bootstrap test (1000 replicates) was shown next to the branches of the consensus tree. Initial tree(s) for the heuristic search were obtained automatically as follows. When the number of common sites was < 100 or less than one fourth of the total number of sites, the maximum parsimony method was employed; otherwise BIONJ method with MCL distance matrix was used. A discrete Gamma distribution was used to model evolutionary rate differences among sites (5 categories). All codon positions were included. All positions with less than 80% site coverage were eliminated. That is, fewer than 20% alignment gaps, missing data, and ambiguous bases were allowed at any position. Evolutionary analyses were conducted in MEGA5 [125].

## Supporting Information

S1 Fig Contingence table comparing the existence of PPs from human genes that contain 3’UTR-embedded Alus with respect to the orientation of these SINEs. (PDF)

S2 Fig Contingence tables comparing the presence of Alu elements inside intronic regions or 5’UTRs of human genes and the existence of PPs from these genes. (PDF)

S3 Fig Contingence tables showing overrepresentation of Alu-like elements inside 3’UTRs of mouse or rat PP parent genes resulting from the combined analysis of mouse and rat data together. (PDF)

S4 Fig Contingence tables showing that B2 elements are not over or underrepresented inside 3’UTRs of mouse or rat PP parent genes. (PDF)

S5 Fig Contingence tables comparing the presence of B1 elements inside intronic regions or 5’UTRs of mouse genes and the existence of PPs from these genes. (PDF)

S6 Fig Sampling analysis to discard the possible effect of transcript length differences between Alu− and Alu+ genes. (PDF)

S7 Fig Sampling analysis to separate the possible effect of the transcript length on the overrepresentation of 3’UTR-embedded Alu elements in PP parent genes. (PDF)

S8 Fig Sampling analysis to separate the possible effect of the GC-content on the overrepresentation of 3’UTR-embedded Alu elements in PP parent genes. (PDF)

S9 Fig Sampling analysis to separate the possible effect of the germline gene expression level on the overrepresentation of 3’UTR-embedded Alu elements in PP parent genes. (PDF)

S10 Fig Phylogenetic trees of CD59, IL17A, and their homologs in the herpesvirus saimiri (HVS). (PDF)

S11 Fig Phylogenetic tree of TYMS and its herpesviral homologs. (PDF)

S12 Fig Alignment of the 3’UTR of CD48 isoform 2 transcript with annotated repeat elements. (PDF)

S1 Table Human gene set. (XLSX)

S2 Table Mouse gene set. (XLSX)

S3 Table Rat gene set. (XLSX)

S4 Table Germline Samples. (PDF)

S5 Table GenBank IDs of the primate host and viral protein sequences aligned. (PDF)

S6 Table IDs of the DNA sequences used in the phylogenetics analysis. (PDF)

S1 File Sampling Perl script. (TXT)

S2 File Amino acid alignments of host/herpesviral homologs. (TXT)
