# Supplementary material for: Novel Role of 3’UTR-Embedded Alu Elements as Facilitators of Processed Pseudogene Genesis and Host Gene Capture by Viral Genomes
Source: PLoS One. 2016 Dec 29;11(12):e0169196. doi: 10.1371/journal.pone.0169196 (PMC5199112; doi:10.1371/journal.pone.0169196)
Supplement: S1 Fig — (PDF) [file pone.0169196.s001.pdf]

***Homo sapiens* genes with 3'UTR-embedded Alu(s)**

		Sense	Antisense	Mix	Total
PPs	–	**1,249** (42.86%) (87.16%)	**1,139** (39.09%) (84.43%)	**526** (18.05%) (81.55%)	2,914
	+	**184** (35.87%) (12.84%)	**210** (40.93%) (15.57%)	**119** (23.20%) (18.45%)	513
	Total	1,433	1,349	645	3,427

χ^2 test P = 0.0030

S1 Fig. Contingence table comparing the existence of PPs from human genes that contain 3'UTR-embedded Alus with respect to the orientation of these SINEs. The genes were grouped in three classes: *sense* (all the 3'UTR-embedded Alu elements have the same sense that the containing gene), *antisense* (all the 3'UTR Alus are in the sense opposite to the gene), and *mix* (3'UTR Alus in both senses). Plus and minus signs on the left mean presence or absence, respectively, of PPs generated from a gene. Numbers in bold are gene counts; total number of genes are also displayed in the right column and the bottom row. Percentages with respect to each total are also shown. P-value of the χ^2 test is also indicated.
