# Supplementary material for: Novel Role of 3’UTR-Embedded Alu Elements as Facilitators of Processed Pseudogene Genesis and Host Gene Capture by Viral Genomes
Source: PLoS One. 2016 Dec 29;11(12):e0169196. doi: 10.1371/journal.pone.0169196 (PMC5199112; doi:10.1371/journal.pone.0169196)
Supplement: S2 Fig — (PDF) [file pone.0169196.s002.pdf]

Homo sapiens **genes**

A

Alu(s) inside introns

		−	+	Total
PPs	−	**2,130** (14.25%) (87.65%)	**12,820** (85.75%) (87.70%)	14,950
	+	**300** (14.30%) (12.35%)	**1798** (85.70%) (12.30%)	2,098
	Total	2,430	14,618	17,048

χ^2 test P = 0.9493

B

5'UTR-embedded Alu

		−	+	Total
PPs	−	**14,655** (98.03%) (87.79%)	**295** (1.97%) (83.33%)	14,950
	+	**2,039** (97.19%) (12.21%)	**59** (2.81%) (16.67%)	2,098
	Total	16,694	354	17,048

χ^2 test P = 0.0116

S2 Fig. Contingence tables comparing the presence of Alu elements inside intronic regions or 5'UTRs of human genes and the existence of PPs from these genes. Plus and minus signs above the tables indicate presence or absence, respectively, of Alu elements inside the introns (**A**) or 5'UTR(s) (**B**) of a gene. Plus and minus signs on the left mean presence or absence, respectively, of PPs generated from a gene. Numbers in bold are gene counts; total number of genes are also displayed in the right column and the bottom row for each table. Percentages with respect to each total are also shown. P-values of the χ^2 test are indicated below each corresponding table.
