# Supplementary material for: Novel Role of 3’UTR-Embedded Alu Elements as Facilitators of Processed Pseudogene Genesis and Host Gene Capture by Viral Genomes
Source: PLoS One. 2016 Dec 29;11(12):e0169196. doi: 10.1371/journal.pone.0169196 (PMC5199112; doi:10.1371/journal.pone.0169196)
Supplement: S3 Fig — (PDF) [file pone.0169196.s003.pdf]

***Mus musculus/Rattus norvegicus* genes**

A

		3'UTR-embedded B1 or B2 −		3'UTR-embedded B1 or B2 +		Total
PPs	−	**10,354** (90.58%)	(78.68%)	**2,806** (86.79%)	(21.32%)	13,160
	+	**1,077** (9.42%)	(71.61%)	**427** (13.21%)	(28.39%)	1,504
	Total	11,431		3,233		14,664

χ^2 test P = 3.7e-10

B

		3'UTR-embedded B1 −		3'UTR-embedded B1 +		Total
PPs	−	**10,828** (90.50%)	(82.28%)	**2,332** (86.37%)	(17.72%)	13,160
	+	**1,136** (9.50%)	(75.53%)	**368** (13.63%)	(24.47%)	1,504
	Total	11,964		2,700		14,664

χ^2 test P = 1.6e-10

S3 Fig. Contingence tables showing overrepresentation of Alu-like elements inside 3’UTRs of mouse or rat PP parent genes resulting from the combined analysis of mouse and rat data together. Plus and minus signs above the tables indicate presence or absence, respectively, of B1 or B2 elements (**A**), or B1 elements alone (**B**) inside the 3’UTR(s) of a gene in whatever of the two species. Plus and minus signs on the left mean presence or absence, respectively, of PPs generated from a gene, whether in mouse or rat. Numbers in bold are gene counts; total number of genes are also displayed in the right column and the bottom row for each table. Percentages with respect to each total are also shown. P-values of the χ^2 test are indicated below each corresponding table.
