# Supplementary material for: Novel Role of 3’UTR-Embedded Alu Elements as Facilitators of Processed Pseudogene Genesis and Host Gene Capture by Viral Genomes
Source: PLoS One. 2016 Dec 29;11(12):e0169196. doi: 10.1371/journal.pone.0169196 (PMC5199112; doi:10.1371/journal.pone.0169196)
Supplement: S4 Fig — (PDF) [file pone.0169196.s004.pdf]

A ***Mus musculus*** **genes**

		3'UTR-embedded B2 (no B1) −	3'UTR-embedded B2 (no B1) +	Total
PPs	−	**15,935** (96.67%) (90.46%)	**549** (3.33%) (90.74%)	16,484
	+	**1,680** (96.77%) (9.54%)	**56** (3.23%) (9.26%)	1,736
	Total	17,615	605	18,220

χ^2 test P = 0.8169

B ***Rattus norvegicus*** **genes**

		3'UTR-embedded B2 (no B1) −	3'UTR-embedded B2 (no B1) +	Total
PPs	−	**18,839** (98.80%) (94.79%)	**228** (1.20%) (93.44%)	19,067
	+	**1,035** (98.48%) (5.21%)	**16** (1.52%) (6.56%)	1,051
	Total	19,874	244	20,118

χ^2 test P = 0.3464

S4 Fig. Contingence tables showing that B2 elements are not over or underrepresented inside 3'UTRs of mouse (**A**) **or rat PP** (**B**) **parent genes**. Plus and minus signs above the tables indicate presence or absence, respectively, of B2 elements alone (in absence of B1 elements) inside the 3'UTR(s) of a gene. Plus and minus signs on the left mean presence or absence, respectively, of PPs generated from a gene. Numbers in bold are gene counts; total number of genes are also displayed in the right column and the bottom row for each table. Percentages with respect to each total are also shown. P-values of the χ^2 test are indicated below each corresponding table.
