# Supplementary material for: Novel Role of 3’UTR-Embedded Alu Elements as Facilitators of Processed Pseudogene Genesis and Host Gene Capture by Viral Genomes
Source: PLoS One. 2016 Dec 29;11(12):e0169196. doi: 10.1371/journal.pone.0169196 (PMC5199112; doi:10.1371/journal.pone.0169196)
Supplement: S5 Fig — (PDF) [file pone.0169196.s005.pdf]

***Mus musculus* genes**

A

		B1(s) inside introns −	B1(s) inside introns +	Total
PPs	−	**3,255** (19.75%) (90.12%)	**13,229** (80.25%) (90.56%)	16,484
PPs	+	**357** (20.56%) (9.88%)	**1379** (79.44%) (9.44%)	1,736
	Total	3,612	14,608	18,220

χ^2 test P = 0.4161

B

		5'UTR-embedded B1 −	5'UTR-embedded B1 +	Total
PPs	−	**16,366** (99.28%) (90.51%)	**118** (0.72%) (85.51%)	16,484
PPs	+	**1,716** (98.85%) (9.49%)	**20** (1.15%) (14.49%)	1,736
	Total	18,082	138	18,220

χ^2 test P = 0.0461

S5 Fig. Contingence tables comparing the presence of B1 elements inside intronic regions or 5'UTRs of mouse genes and the existence of PPs from these genes. Plus and minus signs above the tables indicate presence or absence, respectively, of B1 elements inside the introns (**A**) or 5'UTR(s) (**B**) of a gene. Plus and minus signs on the left mean presence or absence, respectively, of PPs generated from a gene. Numbers in bold are gene counts; total number of genes are also displayed in the right column and the bottom row for each table. Percentages with respect to each total are also shown. P-values of the χ^2 test are indicated below each corresponding table.
