# Supplementary material for: Novel Role of 3’UTR-Embedded Alu Elements as Facilitators of Processed Pseudogene Genesis and Host Gene Capture by Viral Genomes
Source: PLoS One. 2016 Dec 29;11(12):e0169196. doi: 10.1371/journal.pone.0169196 (PMC5199112; doi:10.1371/journal.pone.0169196)
Supplement: S6 Fig — (PDF) [file pone.0169196.s006.pdf]

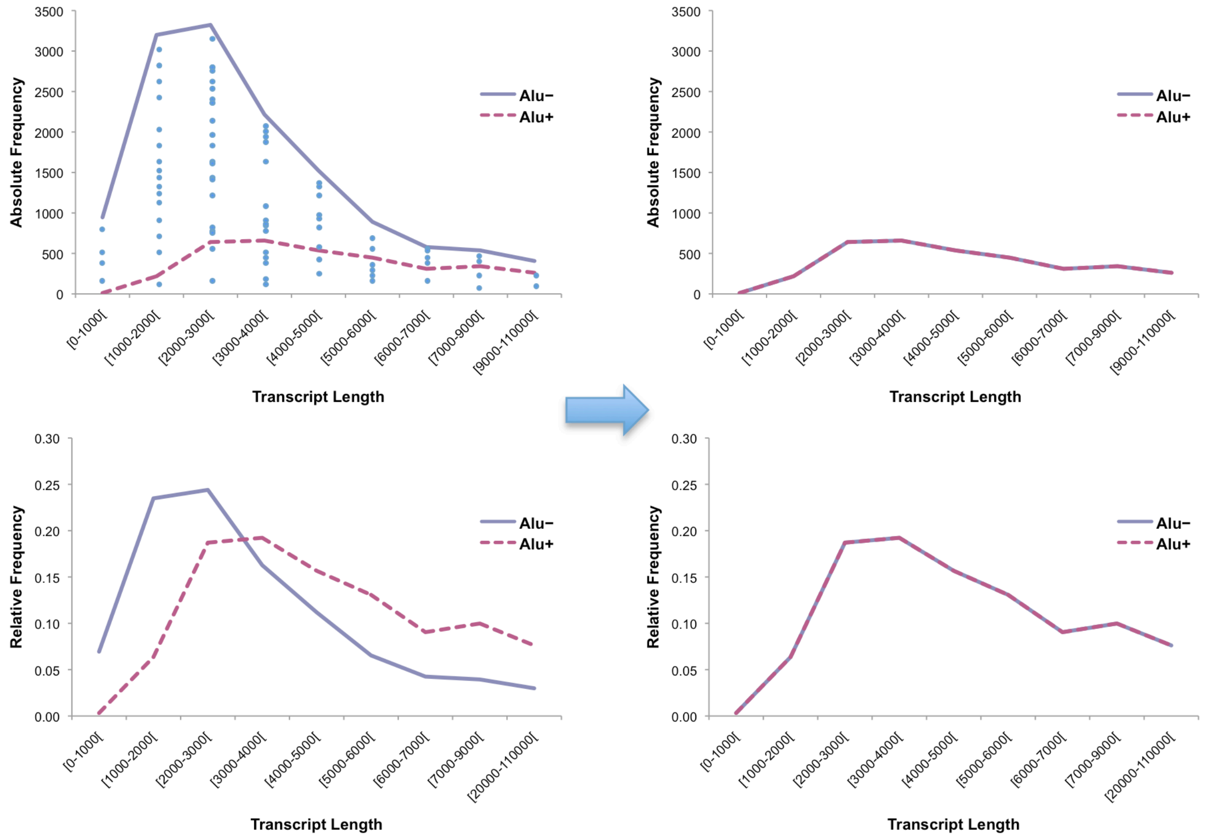

S6 Fig. Sampling analysis to discard the possible effect of transcript length differences between Alu− and Alu+ genes. The genes were grouped into nine bins based on their maximum transcript length. Left plots show the distribution of all Alu− and Alu+ genes (13,621 and 3,427 genes, respectively), in absolute and relative frequency, along the nine bins of transcript length. Right plots show the distribution corresponding to sample 1, after applying random sampling in each bin to generate a sample of Alu− genes with a similar distribution to the Alu+ gene set and the same size (3,427 genes; see Materials and Methods for more details). There are not significant differences between the sampled Alu− and the Alu+ distributions (P = 0.75, Mann-Whitney-Wilcoxon test). Nine more samples were generated using the same method and the results were similar. Blue spots in the upper left graph represent the genes randomly selected in the sampling process.
