# Supplementary material for: Novel Role of 3’UTR-Embedded Alu Elements as Facilitators of Processed Pseudogene Genesis and Host Gene Capture by Viral Genomes
Source: PLoS One. 2016 Dec 29;11(12):e0169196. doi: 10.1371/journal.pone.0169196 (PMC5199112; doi:10.1371/journal.pone.0169196)
Supplement: S7 Fig — (PDF) [file pone.0169196.s007.pdf]

Sample 1

Equal transcript length distribution (P=0.75, MWW test)

		3'UTR-embedded Alu −	3'UTR-embedded Alu +	Total
PPs	−	**3,125** (51.75%) (91.19%)	**2,914** (48.25%) (85.03%)	6,039
PPs	+	**302** (37.06%) (8.81%)	**513** (62.94%) (14.97%)	815
Total		3,427	3,427	6,854

χ^2 test P = 3.4e-15

Sample 2

Equal transcript length distribution (P=0.72, MWW test)

		3'UTR-embedded Alu −	3'UTR-embedded Alu +	Total
PPs	−	**3,131** (51.79%) (91.36%)	**2,914** (48.21%) (85.03%)	6,045
PPs	+	**296** (36.59%) (8.64%)	**513** (63.41%) (14.97%)	809
Total		3,427	3,427	6,854

χ^2 test P = 4.5e-16

Sample 3

Equal transcript length distribution (P=0.72, MWW test)

		3'UTR-embedded Alu −	3'UTR-embedded Alu +	Total
PPs	−	**3,141** (51.87%) (91.65%)	**2,914** (48.13%) (85.03%)	6,055
PPs	+	**286** (35.79%) (8.35%)	**513** (64.21%) (14.97%)	799
Total		3,427	3,427	6,854

χ^2 test P = 1.3e-17

Sample 4

Equal transcript length distribution (P=0.55, MWW test)

		3'UTR-embedded Alu −	3'UTR-embedded Alu +	Total
PPs	−	**3,148** (51.93%) (91.86%)	**2,914** (48.07%) (85.03%)	6,062
PPs	+	**279** (35.23%) (8.14%)	**513** (64.77%) (14.97%)	792
Total		3,427	3,427	6,854

χ^2 test P = 9.5e-19

Sample 5

Equal transcript length distribution (P=0.79, MWW test)

		3'UTR-embedded Alu −	3'UTR-embedded Alu +	Total
PPs	−	**3,151** (51.95%) (91.95%)	**2,914** (48.05%) (85.03%)	6,065
PPs	+	**276** (34.98%) (8.05%)	**513** (65.02%) (14.97%)	789
Total		3,427	3,427	6,854

χ^2 test P = 3.0e-19

Sample 6

Equal transcript length distribution (P=0.66, MWW test)

		3'UTR-embedded Alu −	3'UTR-embedded Alu +	Total
PPs	−	**3,131** (51.79%) (91.36%)	**2,914** (48.21%) (85.03%)	6,045
PPs	+	**296** (36.59%) (8.64%)	**513** (63.41%) (14.97%)	809
Total		3,427	3,427	6,854

χ^2 test P = 4.5e-16

Sample 7

Equal transcript length distribution (P=0.70, MWW test)

		3'UTR-embedded Alu −	3'UTR-embedded Alu +	Total
PPs	−	**3,149** (51.94%) (91.19%)	**2,914** (48.06%) (85.03%)	6,063
PPs	+	**278** (35.15%) (8.81%)	**513** (64.85%) (14.97%)	791
Total		3,427	3,427	6,854

χ^2 test P = 6.5e-19

Sample 8

Equal transcript length distribution (P=0.71, MWW test)

		3'UTR-embedded Alu −	3'UTR-embedded Alu +	Total
PPs	−	**3,135** (51.83%) (91.48%)	**2,914** (48.17%) (85.03%)	6,049
PPs	+	**292** (36.27%) (8.52%)	**513** (63.73%) (14.97%)	805
Total		3,427	3,427	6,854

χ^2 test P = 1.1e-16

Sample 9

Equal transcript length distribution (P=0.69, MWW test)

		3'UTR-embedded Alu −	3'UTR-embedded Alu +	Total
PPs	−	**3,141** (51.87%) (91.65%)	**2,914** (48.13%) (85.03%)	6,055
PPs	+	**286** (35.79%) (8.35%)	**513** (64.21%) (14.97%)	799
Total		3,427	3,427	6,854

χ^2 test P = 1.3e-17

Sample 10

Equal transcript length distribution (P=0.76, MWW test)

		3'UTR-embedded Alu −	3'UTR-embedded Alu +	Total
PPs	−	**3,127** (51.76%) (91.25%)	**2,914** (48.24%) (85.03%)	6,041
PPs	+	**300** (36.90%) (8.75%)	**513** (63.10%) (14.97%)	813
Total		3,427	3,427	6,854

χ^2 test P = 1.8e-15

S7 Fig. Sampling analysis to separate the possible effect of the transcript length on the overrepresentation of 3'UTR-embedded Alu elements in PP parent genes. Ten samples were generated. For each sample, Mann-Whitney-Wilcoxon (MWW) test proved that both gene sets (Alu+ and sampled Alu−) have a similar transcript length distribution and a contingence table showed overrepresentation of 3'UTR-embedded Alu elements in PP parent genes (χ^2 tested). Plus and minus signs above the tables indicate presence or absence, respectively, of Alus inside the 3'UTR(s) of a gene. Plus and minus signs on the left of the tables mean presence or absence, respectively, of PPs generated from a gene. Numbers in bold are gene counts; total number of genes are also displayed in the right column and the bottom row for each table. Percentages with respect to each total are also shown. P-values of the χ^2 test are indicated below each corresponding table. See Materials and Methods for details.
