# Supplementary material for: Novel Role of 3’UTR-Embedded Alu Elements as Facilitators of Processed Pseudogene Genesis and Host Gene Capture by Viral Genomes
Source: PLoS One. 2016 Dec 29;11(12):e0169196. doi: 10.1371/journal.pone.0169196 (PMC5199112; doi:10.1371/journal.pone.0169196)
Supplement: S8 Fig — (PDF) [file pone.0169196.s008.pdf]

Sample 1

Equal GC-content distribution (P=0.99, MWW test)

		3'UTR-embedded Alu −		3'UTR-embedded Alu +		Total
PPs	−	**2,997** (87.45%)	(50.70%)	**2,914** (85.03%)	(49.30%)	5,911
	+	**430** (12.55%)	(45.60%)	**513** (14.97%)	(54.40%)	943
Total		3,427		3,427		6,854

χ^2 test P = 0.0036

Sample 2

Equal GC-content distribution (P=0.96, MWW test)

		3'UTR-embedded Alu −		3'UTR-embedded Alu +		Total
PPs	−	**3,017** (88.04%)	(50.87%)	**2,914** (85.03%)	(49.13%)	5,931
	+	**410** (11.96%)	(44.42%)	**513** (14.97%)	(55.58%)	923
Total		3,427		3,427		6,854

χ^2 test P = 0.0003

Sample 3

Equal GC-content distribution (P=0.94, MWW test)

		3'UTR-embedded Alu −		3'UTR-embedded Alu +		Total
PPs	−	**2,979** (86.93%)	(50.55%)	**2,914** (85.03%)	(49.45%)	5,893
	+	**448** (13.07%)	(46.62%)	**513** (14.97%)	(53.38%)	961
Total		3,427		3,427		6,854

χ^2 test P = 0.0237

Sample 4

Equal GC-content distribution (P=0.96, MWW test)

		3'UTR-embedded Alu −		3'UTR-embedded Alu +		Total
PPs	−	**2,986** (87.13%)	(50.61%)	**2,914** (85.03%)	(49.39%)	5,900
	+	**441** (12.87%)	(46.23%)	**513** (14.97%)	(53.77%)	954
Total		3,427		3,427		6,854

χ^2 test P = 0.0120

Sample 5

Equal GC-content distribution (P=0.93, MWW test)

		3'UTR-embedded Alu −		3'UTR-embedded Alu +		Total
PPs	−	**2,994** (87.37%)	(50.68%)	**2,914** (85.03%)	(49.32%)	5,908
	+	**433** (12.63%)	(45.77%)	**513** (14.97%)	(54.23%)	946
Total		3,427		3,427		6,854

χ^2 test P = 0.0051

Sample 6

Equal GC-content distribution (P=0.99, MWW test)

		3'UTR-embedded Alu −		3'UTR-embedded Alu +		Total
PPs	−	**2,998** (87.48%)	(50.71%)	**2,914** (85.03%)	(49.29%)	5,912
	+	**429** (12.52%)	(45.54%)	**513** (14.97%)	(54.46%)	942
Total		3,427		3,427		6,854

χ^2 test P = 0.0032

Sample 7

Equal GC-content distribution (P=0.99, MWW test)

		3'UTR-embedded Alu −		3'UTR-embedded Alu +		Total
PPs	−	**2,997** (87.45%)	(50.70%)	**2,914** (85.03%)	(49.30%)	5,911
	+	**430** (12.55%)	(45.60%)	**513** (14.97%)	(54.40%)	943
Total		3,427		3,427		6,854

χ^2 test P = 0.0036

Sample 8

Equal GC-content distribution (P=0.97, MWW test)

		3'UTR-embedded Alu −		3'UTR-embedded Alu +		Total
PPs	−	**3,003** (87.63%)	(50.75%)	**2,914** (85.03%)	(49.25%)	5,917
	+	**424** (12.37%)	(45.25%)	**513** (14.97%)	(54.75%)	937
Total		3,427		3,427		6,854

χ^2 test P = 0.0018

Sample 9

Equal GC-content distribution (P=1.00, MWW test)

		3'UTR-embedded Alu −		3'UTR-embedded Alu +		Total
PPs	−	**2,980** (86.96%)	(50.56%)	**2,914** (85.03%)	(49.44%)	5,894
	+	**447** (13.04%)	(46.56%)	**513** (14.97%)	(53.44%)	960
Total		3,427		3,427		6,854

χ^2 test P = 0.0216

Sample 10

Equal GC-content distribution (P=0.96, MWW test)

		3'UTR-embedded Alu −		3'UTR-embedded Alu +		Total
PPs	−	**3,001** (87.57%)	(50.74%)	**2,914** (85.03%)	(49.26%)	5,915
	+	**426** (12.43%)	(45.37%)	**513** (14.97%)	(54.63%)	939
Total		3,427		3,427		6,854

χ^2 test P = 0.0022

S8 Fig. Sampling analysis to separate the possible effect of the GC-content on the overrepresentation of 3’UTR-embedded Alu elements in PP parent genes. Ten samples were generated. For each sample, Mann-Whitney-Wilcoxon (MWW) test proved that both gene sets (Alu+ and sampled Alu−) have a similar GC-content distribution and a contingence table showed overrepresentation of 3’UTR-embedded Alu elements in PP parent genes (χ^2 tested). Plus and minus signs above the tables indicate presence or absence, respectively, of Alus inside the 3’UTR(s) of a gene. Plus and minus signs on the left of the tables mean presence or absence, respectively, of PPs generated from a gene. Numbers in bold are gene counts; total number of genes are also displayed in the right column and the bottom row for each table. Percentages with respect to each total are also shown. P-values of the χ^2 test are indicated below each corresponding table. See Materials and Methods for details.
