# Supplementary material for: Novel Role of 3’UTR-Embedded Alu Elements as Facilitators of Processed Pseudogene Genesis and Host Gene Capture by Viral Genomes
Source: PLoS One. 2016 Dec 29;11(12):e0169196. doi: 10.1371/journal.pone.0169196 (PMC5199112; doi:10.1371/journal.pone.0169196)
Supplement: S9 Fig — (PDF) [file pone.0169196.s009.pdf]

Sample 1

Equal germline gene expression mean distribution (P=0.93, MWW test)

		3'UTR-embedded Alu −		3'UTR-embedded Alu +		Total
PPs	−	**1,588** (87.78%)	(51.03%)	**1,524** (84.25%)	(48.97%)	3,112
	+	**221** (12.22%)	(43.68%)	**285** (15.75%)	(56.32%)	506
Total		1,809		1,809		3,618

χ^2 test P = 0.0022

Sample 2

Equal germline gene expression mean distribution (P=0.91, MWW test)

		3'UTR-embedded Alu −		3'UTR-embedded Alu +		Total
PPs	−	**1,591** (87.95%)	(51.08%)	**1,524** (84.25%)	(48.92%)	3,115
	+	**218** (12.05%)	(43.34%)	**285** (15.75%)	(56.66%)	503
Total		1,809		1,809		3,618

χ^2 test P = 0.0013

Sample 3

Equal germline gene expression mean distribution (P=0.74, MWW test)

		3'UTR-embedded Alu −		3'UTR-embedded Alu +		Total
PPs	−	**1,567** (86.62%)	(50.70%)	**1,524** (84.25%)	(49.30%)	3,091
	+	**242** (13.38%)	(45.92%)	**285** (15.75%)	(54.08%)	527
Total		1,809		1,809		3,618

χ^2 test P = 0.0427

Sample 4

Equal germline gene expression mean distribution (P=0.99, MWW test)

		3'UTR-embedded Alu −		3'UTR-embedded Alu +		Total
PPs	−	**1,590** (87.89%)	(51.06%)	**1,524** (84.25%)	(48.94%)	3,114
	+	**219** (12.11%)	(43.45%)	**285** (15.75%)	(56.55%)	504
Total		1,809		1,809		3,618

χ^2 test P = 0.0015

Sample 5

Equal germline gene expression mean distribution (P=0.95, MWW test)

		3'UTR-embedded Alu −		3'UTR-embedded Alu +		Total
PPs	−	**1,590** (87.89%)	(51.06%)	**1,524** (84.25%)	(48.94%)	3,114
	+	**219** (12.11%)	(43.45%)	**285** (15.75%)	(56.55%)	504
Total		1,809		1,809		3,618

χ^2 test P = 0.0015

Sample 6

Equal germline gene expression mean distribution (P=0.87, MWW test)

		3'UTR-embedded Alu −		3'UTR-embedded Alu +		Total
PPs	−	**1,611** (89.05%)	(51.39%)	**1,524** (84.25%)	(48.61%)	3,135
	+	**198** (10.95%)	(40.99%)	**285** (15.75%)	(59.01%)	483
Total		1,809		1,809		3,618

χ^2 test P = 2.1e-5

Sample 7

Equal germline gene expression mean distribution (P=0.91, MWW test)

		3'UTR-embedded Alu −		3'UTR-embedded Alu +		Total
PPs	−	**1,584** (87.56%)	(50.97%)	**1,524** (84.25%)	(49.03%)	3,108
	+	**225** (12.44%)	(44.12%)	**285** (15.75%)	(55.88%)	510
Total		1,809		1,809		3,618

χ^2 test P = 0.0041

Sample 8

Equal germline gene expression mean distribution (P=0.97, MWW test)

		3'UTR-embedded Alu −		3'UTR-embedded Alu +		Total
PPs	−	**1,585** (87.62%)	(50.98%)	**1,524** (84.25%)	(49.02%)	3,109
	+	**224** (12.38%)	(44.01%)	**285** (15.75%)	(55.99%)	509
Total		1,809		1,809		3,618

χ^2 test P = 0.0035

Sample 9

Equal germline gene expression mean distribution (P=0.95, MWW test)

		3'UTR-embedded Alu −		3'UTR-embedded Alu +		Total
PPs	−	**1,573** (86.95%)	(50.79%)	**1,524** (84.25%)	(49.21%)	3,097
	+	**236** (13.05%)	(45.30%)	**285** (15.75%)	(54.70%)	521
Total		1,809		1,809		3,618

χ^2 test P = 0.0203

Sample 10

Equal germline gene expression mean distribution (P=0.90, MWW test)

		3'UTR-embedded Alu −		3'UTR-embedded Alu +		Total
PPs	−	**1,583** (87.51%)	(50.95%)	**1,524** (84.25%)	(49.05%)	3,107
	+	**226** (12.49%)	(44.23%)	**285** (15.75%)	(55.77%)	511
Total		1,809		1,809		3,618

χ^2 test P = 0.0049

S9 Fig. Sampling analysis to separate the possible effect of the germline gene expression level on the overrepresentation of 3'UTR-embedded Alu elements in PP parent genes. Ten samples were generated. For each sample, Mann-Whitney-Wilcoxon (MWW) test proved that both gene sets (Alu+ and sampled Alu−) have a similar germline gene expression mean distribution and a contingence table showed overrepresentation of 3'UTR-embedded Alu elements in PP parent genes (χ^2 tested). Plus and minus signs above the tables indicate presence or absence, respectively, of Alus inside the 3'UTR(s) of a gene. Plus and minus signs on the left of the tables mean presence or absence, respectively, of PPs generated from a gene. Numbers in bold are gene counts; total number of genes are also displayed in the right column and the bottom row for each table. Percentages with respect to each total are also shown. P-values of the χ^2 test are indicated below each corresponding table. See Materials and Methods for details.
