# Supplementary material for: Novel Role of 3’UTR-Embedded Alu Elements as Facilitators of Processed Pseudogene Genesis and Host Gene Capture by Viral Genomes
Source: PLoS One. 2016 Dec 29;11(12):e0169196. doi: 10.1371/journal.pone.0169196 (PMC5199112; doi:10.1371/journal.pone.0169196)
Supplement: S10 Fig — (PDF) [file pone.0169196.s010.pdf]

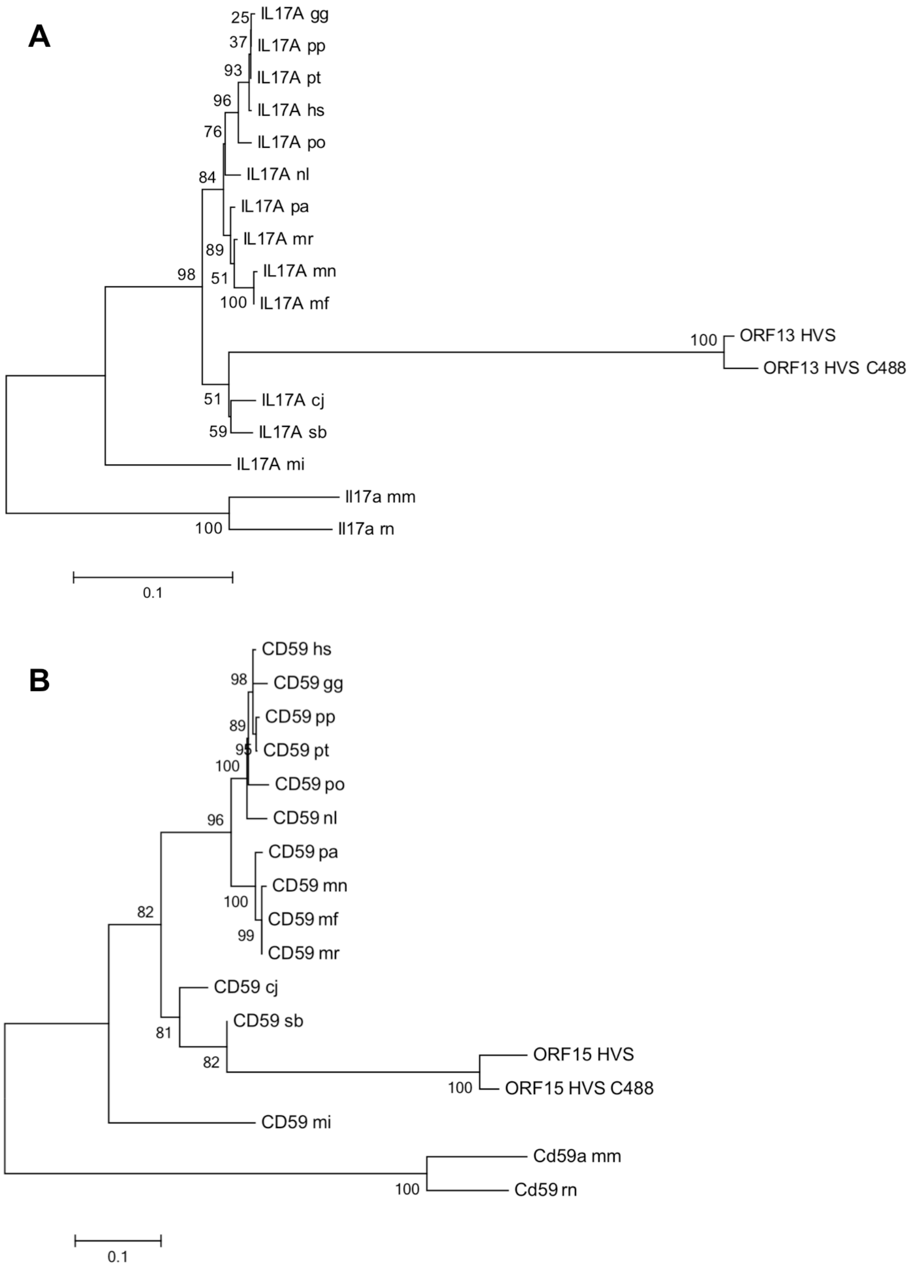

S10 Fig. Phylogenetic trees of *CD59*, *IL17A*, and their homologs in the herpesvirus saimiri (HVS). (**A**) Phylogenetic tree of primate *IL17A* genes and the HVS homolog (*ORF13*). (**B**) Phylogenetic tree of primate *CD59* genes and the HVS homolog (*ORF15*). The phylogenetic analysis used the Maximum Likelihood method based on the HKY85 model. The displayed trees are the resulting bootstrap consensus trees inferred from 1000 replicates. The percentage of replicate trees in which the associated taxa clustered together in the bootstrap test is shown next to the branches. The branch lengths indicate the number of substitutions per site at the scale shown below the tree. The 13 primate species considered were: *Homo sapiens* (hs), *Pan paniscus* (pp), *Pan troglodytes* (pt), *Gorilla gorilla* (gg), *Pongo abelii* (po), *Nomascus leucogenys* (nl), *Macaca fascicularis* (mf), *Macaca nemestrina* (mn), *Macaca mulatta* (mr), *Papio anubis* (pa), *Callithrix jacchus* (cj), *Saimiri boliviensis* (sb), and *Microcebus murinus* (mi). In addition to the normal HVS strain (GenBank NC_001350), the HVS strain C488 (GenBank AJ410493) was also included. *Mus musculus* (mm) and *Rattus norvegicus* (rn) ortologs were used as an outgroup.
