# Supplementary material for: Novel Role of 3’UTR-Embedded Alu Elements as Facilitators of Processed Pseudogene Genesis and Host Gene Capture by Viral Genomes
Source: PLoS One. 2016 Dec 29;11(12):e0169196. doi: 10.1371/journal.pone.0169196 (PMC5199112; doi:10.1371/journal.pone.0169196)
Supplement: S11 Fig — (PDF) [file pone.0169196.s011.pdf]

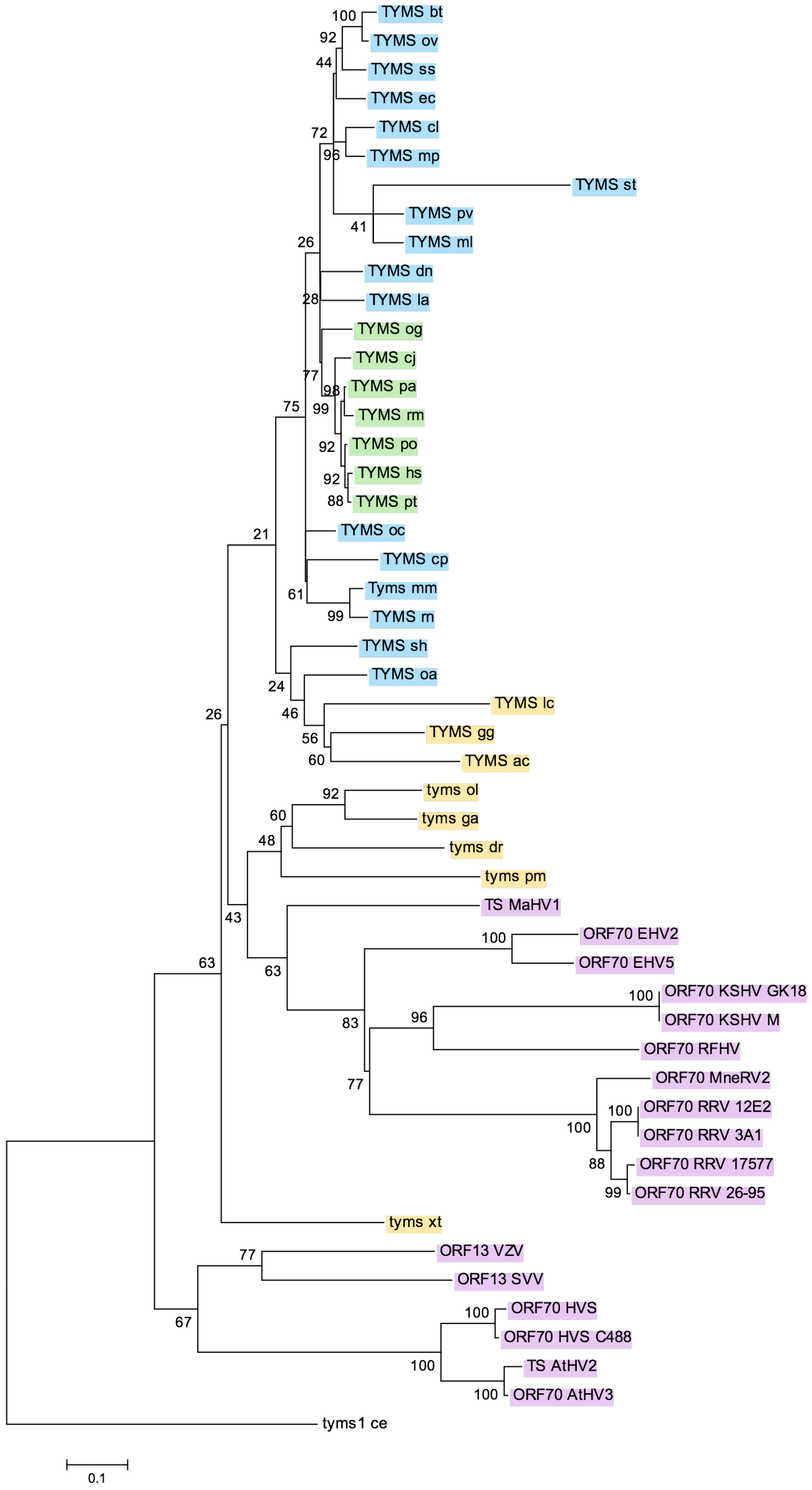
TYMS bt
TYMS ov
TYMS ss
TYMS ec
TYMS cl
TYMS mp
TYMS st
TYMS pv
TYMS ml
TYMS dn
TYMS la
TYMS og
TYMS cj
TYMS pa
TYMS rm
TYMS po
TYMS hs
TYMS pt
TYMS oc
TYMS cp
Tyms mm
TYMS rn
TYMS sh
TYMS oa
TYMS lc
TYMS gg
TYMS ac
tyms ol
tyms ga
tyms dr
tyms pm
TS MaHV1
ORF70 EHV2
ORF70 EHV5
ORF70 KSHV GK18
ORF70 KSHV M
ORF70 RFHV
ORF70 MneRV2
ORF70 RRV 12E2
ORF70 RRV 3A1
ORF70 RRV 17577
ORF70 RRV 26-95
tyms xt
ORF13 VZV
ORF13 SVV
ORF70 HVS
ORF70 HVS C488
TS AtHV2
ORF70 AtHV3
tyms1 ce
0.1

S11 Fig. Phylogenetic tree of *TYMS* and its herpesviral homologs. The phylogenetic analysis used the Maximum Likelihood method based on the HKY85 model. The displayed tree is the resulting bootstrap consensus tree inferred from 1000 replicates. The percentage of replicate trees in which the associated taxa clustered together in the bootstrap test is shown next to the branches. The branch lengths indicate the number of substitutions per site at the scale shown below the tree. The primate species considered (in green) were: *Homo sapiens* (hs), *Pan troglodytes* (pt), *Pongo abelii* (po), *Macaca mulatta* (rm), *Papio anubis* (pa), *Callithrix jacchus* (cj), and *Otolemur garnettii* (og). Other mammal species included (in blue) were: *Bos taurus* (bt), *Ovis aries* (ov), *Sus scrofa* (ss), *Equus caballus* (ec), *Canis lupus familiaris* (cl), *Mustela putorius furo* (mp), *Spermophilus tridecemlineatus* (st), *Pteropus vampyrus* (pv), *Myotis lucifugus* (ml), *Loxodonta africana* (la), *Dasypus novemcinctus* (dn), *Oryctolagus cuniculus* (oc), *Cavia porcellus* (cp), *Mus musculus* (mm), *Rattus norvegicus* (rn), *Sarcophilus harrisii* (sh), and *Ornithorhynchus anatinus* (oa). Other vertebrates species (in yellow) were also considered: *Latimeria chalumnae* (lc), *Gallus gallus* (gg), *Anolis carolinensis* (ac), *Oryzias latipes* (ol), *Gasterosteus aculeatus* (ga), *Danio rerio* (dr), *Petromyzon marinus* (pm), and *Xenopus tropicalis* (xt). The herpesviruses included in the analysis (in purple) were: Macropodid herpesvirus 1 (MaHV1), Equid herpesvirus 2 (EHV2), Equid herpesvirus 5 (EHV5), Kaposi's sarcoma-associated herpesvirus (KSHV, strain GK18 and type M), Retroperitoneal fibromatosis-associated herpesvirus (RFHV), Macaca nemestrina rhadinovirus 2 (MneRV2), Rhesus macaque rhadinovirus (RRV, strains 12E2, 3A1, 17577, and 26-95), Varicella-zoster virus (VZV), Simian varicella virus (SVV), Herpesvirus saimiri (HVS, normal strain and strain C488), Ateline herpesvirus 2 (AtHV2), and Ateline herpesvirus 3 (AtHV3). *Caenorhabditis elegans* (ce) ortolog (tyms1) was used as an outgroup.
