# Supplementary material for: Novel Role of 3’UTR-Embedded Alu Elements as Facilitators of Processed Pseudogene Genesis and Host Gene Capture by Viral Genomes
Source: PLoS One. 2016 Dec 29;11(12):e0169196. doi: 10.1371/journal.pone.0169196 (PMC5199112; doi:10.1371/journal.pone.0169196)
Supplement: S4 Table — (PDF) [file pone.0169196.s016.pdf]

S4 Table. Germline Samples.

OVARY__2_A	FETAL.OVARY.12WK__11_A
TESTIS__2_A	FETAL.OVARY.12.9WK__11_A
TESTIS__3_A	FETAL.OVARY.13.6WK__11_A
TESTIS__3_B	FETAL.OVARY.13.6WK__11_B
OVARY__3_A	FETAL.OVARY.13.9WK__11_A
TESTIS.GERMCELL__3_A	FETAL.OVARY.14.4WK__11_A
TESTIS.SEMINIFEROUS.TUBULE__3_A	FETAL.OVARY.16.1WK__11_A
TESTIS.GERMCELL__3_B	FETAL.OVARY.16.4WK__11_A
TESTIS.SEMINIFEROUS.TUBULE__3_B	FETAL.OVARY.16.9WK__11_A
OVARY__3_B	FETAL.OVARY.16.9WK__11_B
TESTIS__5_G	FETAL.OVARY.17.1WK__11_A
TESTIS__5_H	FETAL.OVARY.18.1WK__11_A
TESTIS__5_I	FETAL.TESTIS.9WK__11_A
TESTIS__5_J	FETAL.TESTIS.9.1WK__11_A
TESTIS__5_K	FETAL.TESTIS.9.9WK__11_A
TESTIS__5_L	FETAL.TESTIS.11WK__11_A
TESTIS.SEMINIFEROUS.TUBULE__9_A	FETAL.TESTIS.11WK__11_B
TESTIS.SEMINIFEROUS.TUBULE__9_B	FETAL.TESTIS.11.7WK__11_A
TESTIS.SPERMATID__9_A	FETAL.TESTIS.12WK__11_A
TESTIS.SPERMATID__9_B	FETAL.TESTIS.12.6WK__11_A
TESTIS.SPERMATOCYTE__9_A	FETAL.TESTIS.13.6WK__11_A
TESTIS.SPERMATOCYTE__9_B	FETAL.TESTIS.13.9WK__11_A
TESTIS__9_A	FETAL.TESTIS.13.9WK__11_B
TESTIS__9_B	FETAL.TESTIS.16.1WK__11_A
OOCYTE__10_A	FETAL.TESTIS.16.1WK__11_B
OOCYTE__10_B	FETAL.TESTIS.17.2WK__11_A
OOCYTE__10_C	FETAL.TESTIS.18.6WK__11_A
FETAL.OVARY.9.1WK__11_A	FETAL.TESTIS.18.9WK__11_A
FETAL.OVARY.9.6WK__11_A	FETAL.TESTIS.19.9WK__11_A
FETAL.OVARY.9.6WK__11_B	TESTIS.SPERMATOGONIA__8_A
FETAL.OVARY.9.6WK__11_C	TESTIS.SPERMATOGONIA__8_B
FETAL.OVARY.11WK__11_A	TESTIS.SPERMATOGONIA__8_C

These are the germline tissue samples from the study of McVicker and Green [65] that we used in our study to estimate human germline gene expression.
