# Supplementary material for: Novel Role of 3’UTR-Embedded Alu Elements as Facilitators of Processed Pseudogene Genesis and Host Gene Capture by Viral Genomes
Source: PLoS One. 2016 Dec 29;11(12):e0169196. doi: 10.1371/journal.pone.0169196 (PMC5199112; doi:10.1371/journal.pone.0169196)
Supplement: S5 Table — (PDF) [file pone.0169196.s017.pdf]

S5 Table. GenBank IDs of the primate host and viral protein sequences aligned.

Host Gene	Host Species	Host Protein ID	Viral Gene	Viral Species	Viral Protein ID
IL10	*Macaca fascicularis*	XP_005540710	*BCRF1*	Lymphocryptovirus Macaca	ALF03208
IL10	*Macaca mulatta*	NP_001038192	*BCRF1*	Macacine Herpesvirus 4	YP_067941
IL10	*Homo sapiens*	NP_000563	*BCRF1*	Epstein-Barr Virus (EBV)	YP_001129439
IL10	*Papio anubis*	XP_003893246	*vIL10*	Papiine Herpesvirus 1	AAF23949
DHFR	*Saimiri boliviensis*	XP_010340850	*ORF2*	Herpesvirus Saimiri (HVS)	NP_040203
DHFR	*Macaca nemestrina*	XP_011728325	*ORF2*	Macaca nemestrina Rhadinovirus 2 (MneRV2)	AJE29641
SLAMF6	*Saimiri boliviensis*	–[a]	*S1*	Squirrel Monkey Cytomegalovirus (SMCMV)	YP_004940177
IL17A	*Saimiri boliviensis*	XP_003923160	*ORF13*	Herpesvirus Saimiri (HVS)	NP_040215
TYMS	*Macaca mulatta*	NP_001182436	*ORF13*	Simian Varicella Virus (SVV)	NP_077428
TYMS	*Aotus nancymaae*	XP_012300458	*ORF70*	Ateline Herpesvirus 3 (AtHV3)	NP_048042
TYMS	*Saimiri boliviensis*	XP_003924847	*ORF70*	Herpesvirus Saimiri (HVS)	NP_040272
TYMS	*Homo sapiens*	NP_001062	*ORF13*	Varicella Zoster Virus (VZV)	AKG58489
TYMS	*Homo sapiens*	NP_001062	*ORF70*	Kaposi's Sarcoma-associated Herpesvirus (KSHV)	ALH44776
TYMS	*Macaca mulatta*	NP_001182436	*ORF70*	Rhesus Monkey Rhadinovirus (RRV)	AAF59999
TYMS	*Macaca nemestrina*	XP_011711015	*ORF70*	Macaca nemestrina Rhadinovirus 2 (MneRV2)	AJE29652
TYMS	*Macaca nemestrina*	XP_011711015	*ORF70*	Retroperitoneal Fibromatosis-associated Herpesvirus (RFHV)	AGY30693
CD59	*Saimiri boliviensis*	XP_010331578	*ORF15*	Herpesvirus Saimiri (HVS)	NP_040217
LOC101037697	*Saimiri boliviensis*	XP_010335696	*S28*	Squirrel Monkey Cytomegalovirus (SMCMV)	YP_004940312
LY9	*Aotus nancymaae*	XP_012305076	*A33*	Owl Monkey Cytomegalovirus (OMCMV)	YP_004940161
CD48	*Aotus nancymaae*	XP_012305080	*A43*	Owl Monkey Cytomegalovirus (OMCMV)	YP_004940172

[a]*S. boliviensis* SLAMF6 protein sequence was predicted from the genome. As this region is not completely sequenced, there are 52 amino acids missed that were extrapolated from the *A. nancymaae* SLAMF6 protein (XP_012305082).
