# Supplementary material for: Novel Role of 3’UTR-Embedded Alu Elements as Facilitators of Processed Pseudogene Genesis and Host Gene Capture by Viral Genomes
Source: PLoS One. 2016 Dec 29;11(12):e0169196. doi: 10.1371/journal.pone.0169196 (PMC5199112; doi:10.1371/journal.pone.0169196)
Supplement: S6 Table — (PDF) [file pone.0169196.s018.pdf]

S6 Table. IDs of the DNA sequences used in the phylogenetics analysis.

Species	Gene	GenBank or Ensembl ID
IL17A phylogenetic tree		
Homo sapiens	*IL17A*	NM_002190
Pan paniscus	*IL17A*	XM_003833162
Pan troglodytes	*IL17A*	XM_527408
Gorilla gorilla	*IL17A*	XM_004044183
Pongo abelii	*IL17A*	XM_002816996
Nomascus leucogenys	*IL17A*	XM_003254163
Macaca fascicularis	*IL17A*	XM_005552759
Macaca nemestrina	*IL17A*	XM_011756846
Macaca mulatta	*IL17A*	XM_001106391
Papio anubis	*IL17A*	XM_003897728
Callithrix jacchus	*IL17A*	XM_002746684
Saimiri boliviensis	*IL17A*	XM_003923111
Microcebus murinus	*IL17A*	XM_012760842
Mus musculus	*Il17a*	NM_010552
Rattus norvegicus	*Il17a*	NM_001106897
Herpesvirus saimiri (HVS)	*ORF13*	NC_001350
Herpesvirus saimiri (HVS) strain C488	*ORF13*	AJ410493
CD59 phylogenetic tree		
Homo sapiens	*CD59*	NM_203330
Pan paniscus	*CD59*	XM_008955860
Pan troglodytes	*CD59*	XM_009460159
Gorilla gorilla	*CD59*	XM_004050913
Pongo abelii	*CD59*	NM_001133389
Nomascus leucogenys	*CD59*	XM_003254387
Macaca fascicularis	*CD59*	NM_001283527
Macaca nemestrina	*CD59*	XM_011724034
Macaca mulatta	*CD59*	NM_001261705
Papio anubis	*CD59*	XM_009186334
Callithrix jacchus	*CD59*	XM_002755162
Saimiri boliviensis	*CD59*	XM_010333276
Microcebus murinus	*CD59*	XM_012739886
Mus musculus	*Cd59*	NM_001111060
Rattus norvegicus	*Cd59*	NM_012925
Herpesvirus saimiri (HVS)	*ORF15*	NC_001350
Herpesvirus saimiri (HVS) strain C488	*ORF15*	AJ410493
TYMS phylogenetic tree		
Homo sapiens	*TYMS*	NM_001071
Pan troglodytes	*TYMS*	NM_001246582
Pongo abelii	*TYMS*	ENSPPYT00000033303
Macaca mulatta	*TYMS*	NM_001195507
Papio anubis	*TYMS*	ENSPANT00000022270
Callithrix jacchus	*TYMS*	ENSCJAT00000000247
Otolemur garnettii	*TYMS*	ENSOGAT00000033315
Bos taurus	*TYMS*	NM_001037816
Ovis aries	*TYMS*	ENSOART00000010195
Sus scrofa	*TYMS*	NM_001243579
Equus caballus	*TYMS*	ENSECAT00000024973
Canis lupus familiaris	*TYMS*	NM_001252174

S6 Table. IDs of the DNA sequences used in the phylogenetics analysis. (cont.)

Species	Gene	GenBank or Ensembl ID
Mustela putorius furo	*TYMS*	ENSMPUT00000005469
Spermophilus tridecemlineatus	*TYMS*	ENSSTOT00000000908
Pteropus vampyrus	*TYMS*	ENSPVAT00000000700
Myotis lucifugus	*TYMS*	ENSMLUT00000009583
Loxodonta africana	*TYMS*	ENSLAFT00000014909
Dasypus novemcinctus	*TYMS*	ENSDNOT00000043823
Oryctolagus cuniculus	*TYMS*	ENSOCUT00000008664
Cavia porcellus	*TYMS*	ENSCPOT00000015726
Mus musculus	*Tyms*	NM_021288
Rattus norvegicus	*Tyms*	NM_019179
Sarcophilus harrisii	*TYMS*	ENSSHAT00000003568
Ornithorhynchus anatinus	*TYMS*	ENSOANT00000018418
Latimeria chalumnae	*TYMS*	ENSLACT00000018651
Gallus gallus	*TYMS*	ENSGALT00000024028
Anolis carolinensis	*TYMS*	ENSACAT00000008476
Oryzias latipes	*tyms*	ENSORLT00000013877
Gasterosteus aculeatus	*tyms*	ENSGACT00000005429
Danio rerio	*tyms*	NM_131760
Petromyzon marinus	*tyms*	ENSPMAT00000007617
Xenopus tropicalis	*tyms*	NM_001079384
Caenorhabditis elegans	*tyms1*	NM_059131
Macropodid herpesvirus 1 (MaHV1)	*TS*	AF188480
Equid herpesvirus 2 (EHV2)	*ORF70*	NC_001650
Equid herpesvirus 5 (EHV5)	*ORF70*	NC_026421
Kaposi's sarcoma-associated herpesvirus (KSHV) strain GK18	*ORF70*	NC_009333
Kaposi's sarcoma-associated herpesvirus (KSHV) type M	*ORF70*	U75698
Retroperitoneal fibromatosis-associated herpesvirus (RFHV)	*ORF70*	KF703446
Macaca nemestrina rhadinovirus 2 (MneRV2)	*ORF70*	KP265674
Rhesus macaque rhadinovirus (RRV) strain 12E2	*ORF70*	JN885137
Rhesus macaque rhadinovirus (RRV) strain 3A1	*ORF70*	JN885136
Rhesus macaque rhadinovirus (RRV) strain 17577	*ORF70*	AF083501
Rhesus macaque rhadinovirus (RRV) strain 26-95	*ORF70*	AF210726
Varicella-zoster virus (VZV)	*ORF13*	NC_001348
Simian varicella virus (SVV)	*ORF13*	NC_002686
Herpesvirus saimiri (HVS)	*ORF70*	NC_001350
Herpesvirus saimiri (HVS) strain C488	*ORF70*	AJ410493
Ateline herpesvirus 2 (AtHV2)	*TS*	M22036
Ateline herpesvirus 3 (AtHV3)	*ORF70*	NC_001987
