# Supplementary material for: Novel Role of 3’UTR-Embedded Alu Elements as Facilitators of Processed Pseudogene Genesis and Host Gene Capture by Viral Genomes
Source: PLoS One. 2016 Dec 29;11(12):e0169196. doi: 10.1371/journal.pone.0169196 (PMC5199112; doi:10.1371/journal.pone.0169196)
Supplement: S12 Fig — (PDF) [file pone.0169196.s012.pdf]

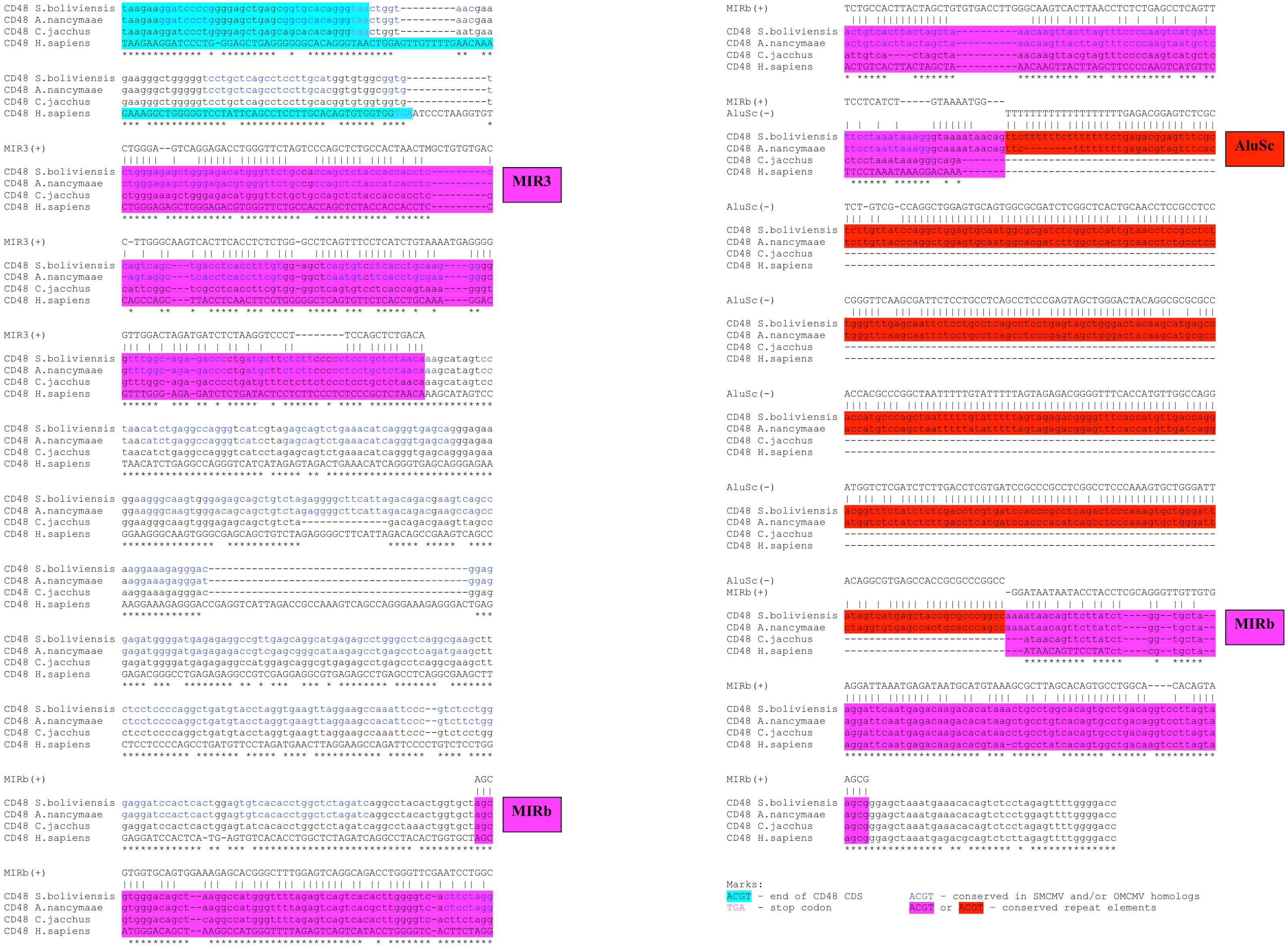

S12 Fig. Alignment of the 3'UTR of CD48 isoform 2 transcript with annotated repeat elements. NW monkey CD48 transcripts were predicted from human CD48 isoform 2 transcript (RefSeq NM_001256030, in uppercase) using the genomic sequence of *Saimiri boliviensis*, *Aotus nancymaae*, and *Callithrix jacchus*.
